# Phenotypic Antimicrobial Resistance of Some Bacterial Strains Isolated from Red Foxes (*Vulpes vulpes*) in Western Romania

**DOI:** 10.3390/antibiotics15020167

**Published:** 2026-02-04

**Authors:** Alex-Cristian Moza, Iulia-Maria Bucur, Kalman Imre, Sebastian Alexandru Popa, Alexandra Adriana Grigoreanu, Ana-Maria Plotuna, Andrei Alexandru Ivan, Narcisa Geanina Mederle, Andreea-Talida Tîrziu, Emil Tîrziu

**Affiliations:** 1 Faculty of Veterinary Medicine, University of Life Sciences “King Mihai I” from Timisoara, Calea Aradului 119, 300645 Timisoara, Romania; alex.moza@usvt.ro (A.-C.M.); kalmanimre@usvt.ro (K.I.); sebastian.popa@usvt.ro (S.A.P.); alexandra.grigoreanu@usvt.ro (A.A.G.); anamaria.plotuna@usvt.ro (A.-M.P.); andreialexandru.ivan.fmv@usvt.ro (A.A.I.); narcisamederle@usvt.ro (N.G.M.); emiltirziu@usvt.ro (E.T.); 2 Ophtalmology Department, Faculty of Medicine, “Victor Babes” University of Medicine and Pharmacy, Piata Eftimie Murgu 2, 300041 Timisoara, Romania; andreea.tirziu@umft.ro

**Keywords:** red foxes, multidrug resistance, European wildlife, One Health

## Abstract

Background/Objectives: Recent investigations point to red foxes (*Vulpes vulpes*) as a very potent sentinel species for monitoring the dissemination of antimicrobial bacteria in wildlife habitats. Methods: This study investigated antimicrobial resistance in red foxes from 16 hunting grounds (peri-urban and peri-rural) in western Romania, between 2022 and 2024, in order to evaluate the species as “One Health” sentinels at the wildlife–human–animal interface. During this period, 137 bacterial strains previously identified from 216 samples were phenotypically tested using both the Kirby–Bauer disk diffusion method and the Vitek 2 Compact system. Results: Among the Gram-negative isolates, particularly *Escherichia coli* and *Salmonella enterica*, notable antimicrobial resistance and multidrug-resistant (MDR) phenotypes were observed, including resistance to third-generation cephalosporins (ceftazidime) and reduced susceptibility to carbapenems. Resistance patterns observed in *Proteus* spp. largely reflected intrinsic resistance traits. Methicillin-resistant and MDR staphylococci (*Staphylococcus aureus*, *S. pseudintermedius* and *S. sciuri*) were detected in both peri-urban and peri-rural hunting grounds, with higher frequencies observed in peri-rural areas. Although MDR prevalence was slightly higher in peri-urban compared to peri-rural sites, no statistically significant association was identified between area of isolation and antimicrobial resistance or MDR status. Antimicrobial susceptibility results obtained by disk diffusion and the Vitek 2 Compact system showed a high level of concordance for antibiotics tested in common. Conclusions: Overall, these findings support the use of red foxes as effective One Health sentinels for monitoring environmental antimicrobial resistance occurrence across wildlife, domestic animals, and human-impacted habitats.

## 1. Introduction

Well before the discovery of antibiotics and their development to treat infectious diseases, antimicrobial resistance had naturally developed due to the presence of environmental microorganisms, which are able to synthetize natural antibiotics [1]. However, during the past several decades, the selection and spread of multidrug-resistant bacteria have been accelerated by the extensive and frequently inappropriate use of antibiotics in human medicine, veterinary care, and agriculture [2,3,4,5].

The most effective means of combating bacterial infections is represented by antibiotics. Presently, The World Health Organization (WHO) has recognized antimicrobial resistance (AMR) as one of the top ten emerging risks of the twenty-first century, making it one of the greatest threats to global public health [2]. Furthermore, the “One Health” (OH) research has acknowledged the link between humans, animals, and the environment, emphasizing how AMR affects all three groups collectively [6,7].

Although most wildlife species inhabit environments distant from human activity, certain species, such as the red fox, have successfully adapted to peri-urban and urban areas, where they coexist with humans and domestic animals. Unlike livestock or companion animals, wildlife is generally not subjected to direct antimicrobial selection pressure through therapeutic use, except in rare circumstances. Consequently, the detection of antimicrobial-resistant bacteria in wildlife is largely attributed to indirect exposure pathways, including interactions with humans and domestic animals and environmental contamination by anthropogenic waste [7,8,9,10,11].

In this context, an increasing number of studies recognize wildlife as an important reservoir of antimicrobial-resistant (AMR) zoonotic pathogens. Both phenotypic and genotypic resistance have been documented in bacterial species of major clinical relevance, such as *Escherichia coli*, *Salmonella* spp., methicillin-resistant *Staphylococcus aureus* (MRSA), *Listeria* spp., and *Enterococcus* spp., within wild animal populations, indicating that antimicrobial resistance is no longer confined to human and domestic animal environments [9,12,13,14]. Despite substantial advances in AMR surveillance in humans and livestock, comparable data from wildlife species remain limited, underscoring a significant knowledge gap [15,16].

The red fox (*Vulpes vulpes*), the most widely distributed species of wild canid in Europe, represents a particularly suitable model for addressing this gap [3]. With its diverse diet and extensive mobility, it constitutes a significant ecological vector capable of acquiring and transmitting antimicrobial-resistant (AMR) bacteria across habitats. Evidence shows that foxes can acquire AMR bacteria and antibiotic residues from contaminated soil, food, and waste in human-dominated environments, establishing a bidirectional flow of resistance [1,17]. Their growing proximity to rural and urban areas increases the likelihood of direct and indirect contact with humans and domestic animals. Consequently, the red fox has been proposed as a sentinel species highly effective for monitoring the dissemination of AMR bacteria in wildlife ecosystems. This is supported by studies which report the fox’s capacity to carry significant bacterial pathogens. For foodborne agents like *Salmonella* spp., *Listeria monocytogenes*, and Shiga toxin-producing *E. coli* (STEC), foxes primarily serve as environmental sentinels, indicating ecosystem contamination. However, for antimicrobial-resistant bacteria critical in healthcare settings, such as Extended-Spectrum Beta-Lactamase (ESBL)-producing *E. coli*, MRSA, and vancomycin-resistant *Enterococcus faecium* (VRE), foxes can act as active vehicles for dissemination [4,7,15,18,19,20,21].

Although adult red foxes often maintain relatively stable home ranges, their adaptability and occasional extra-territorial movements, particularly during dispersal, enable interactions between anthropogenic and natural habitats, which may facilitate the dissemination and persistence of bacteria, including antimicrobial-resistant strains, across ecosystem boundaries. In order to fully comprehend the complex relationship between environmental, animal, and human health, and to develop resulting strategies to control further contamination, it is mandatory for us to place these findings into the One Health concept [15,22,23,24,25].

In this context, acquiring new data regarding the fight against one of the most important global threats has become essential for both human and veterinary medicine, as antimicrobial resistance is a persistently expanding issue.

In this context, the aim of the study was to examine populations of red fox in western Romania, in order to evaluate the levels of phenotypic antimicrobial resistance in multiple species of Gram-negative and Gram-positive bacteria, based on samples isolated from the oral cavities and rectums of *Vulpes vulpes* individuals of different ages and sex, hunted from multiple hunting grounds.

## 2. Results

To ensure a more accurate visualization of the evolution of antibiotic resistance in bacteria isolated from red foxes, the results across the 16 hunting grounds were grouped as peri-urban and peri-rural, according to the corresponding county and the geographic proximity of the sites. Therefore, the groups were defined as follows: from Timiș County, (1) Buziaș and Moșnița hunting grounds, (2) Oloșag, Boldur and Sacoșul Mare, (3) Nădrag and Surduc, (4) Făget, Traian Vuia and Margina, and (5) Belinț-Chizătău, Paniova and Valea Lungă; from Hunedoara County, Silvaș and Zeicani hunting grounds; and from Arad County, the Crocna-Zimbru hunting ground.

### 2.1. Antimicrobial Susceptibility Results for Some Gram-Negative Bacterial Species Acquired with the Disk Diffusion Method

In order to evaluate the phenotypic antimicrobial resistance profiles of wildlife-associated bacteria, a total of 137 isolates were analyzed through the disk diffusion method. These included 73 Gram-negative strains and 64 Gram-positive isolates, all belonging to the genus *Staphylococcus*. To ensure accuracy in data interpretation and to avoid the inflation of resistance rates, all isolates initially classified as “susceptible, increased exposure” (I) were considered susceptible (S) at the tested antibiotic concentrations, following the recommendations of the European Committee on Antimicrobial Susceptibility Testing (EUCAST) and Clinical and Laboratory Standards Institute (CLSI) guidelines. Antimicrobials for which a species is intrinsically resistant were not described based on susceptibility testing and are indicated as IR (intrinsic resistance) according to EUCAST guidelines. These isolates were excluded from the calculation of resistance rates [26,27].

#### 2.1.1. Peri-Urban Hunting Grounds

##### Buziaș and Moșnița Hunting Grounds (Timiș County)

In order to assess the susceptibility of Gram-negative species to antimicrobials, samples collected from the Buziaș and Moșnița hunting grounds were analyzed. A total of twenty-two strains were included, consisting of *Escherichia coli* (n = 16), *Enterobacter* spp. (n = 1), *Proteus mirabilis* (n = 3), and *Salmonella enterica* subsp. *enterica* (n = 2).

According to the antimicrobial susceptibility analysis, these *E. coli* strains (n = 16) exhibited their highest resistance when tested against ceftazidime (50%), followed by amikacin (50%), amoxicillin, and ampicillin (44%). By contrast, the greatest susceptibility was observed to nitrofurantoin and trimethoprim–sulfamethoxazole (100%), followed by cephalexin (94%), nalidixic acid (94%), chloramphenicol (88%), and tetracycline (88%). The *Enterobacter* spp. isolate showed resistance only towards ciprofloxacin (Table 1).

Regarding the *P. mirabilis* strains, one isolate was non-susceptible to ceftazidime, cephalexin, and all aminoglycosides. Both *Salmonella* strains exhibited resistance to at least 11 of the tested antimicrobial drugs. The first strain remained susceptible to cephalexin, amikacin, and chloramphenicol, whereas the second strain was susceptible to ampicillin, amoxicillin, and tetracycline.

Additionally, it should be noted that all strains of *Salmonella enterica* subsp. *enterica*, as well as four (25%) of the *E. coli* isolates, were found to be multidrug resistant (MDR) (Table 1).

##### Făget, Traian Vuia, and Margina Hunting Grounds (Timiș County)

According to the data shown in Table 1, the eight *E. coli* strains isolated from the red foxes exhibited 50% resistance against cefoxitin, amikacin, and tobramycin. The highest susceptibility rates were recorded for trimethoprim–sulfamethoxazole (100%), followed by ceftazidime, cephalexin, imipenem, nalidixic acid, and nitrofurantoin (87.5%).

Multidrug resistance was confirmed in three out of eight *E. coli* isolates (37.5%).

#### 2.1.2. Peri-Rural Hunting Grounds

##### Oloșag, Boldur, and Sacoșul Mare Hunting Grounds (Timiș County)

The results for the Gram-negative strains that were isolated from these hunting areas were mostly in line with those obtained from the Buziaș and Moșnița hunting grounds. Among the *E. coli* isolates (n = 15), high resistance was again observed against ceftazidime, ampicillin, and amoxicillin (40%), followed by imipenem and amikacin (33%). Conversely, there was evidence of high susceptibility to trimethoprim–sulfamethoxazole (100%) and nitrofurantoin and chloramphenicol (93%), as well as nalidixic acid (87%) and cefoxitin, cephalexin, gentamicin, tobramycin, and tetracycline (80%) (Table 2).

The six *P. mirabilis* isolates were responsive to most of the tested antibiotics, while a couple of strains showed some resistance to trimethoprim–sulfamethoxazole, ceftazidime, and chloramphenicol. Moreover, the two *Shigella sonnei* isolates showed 100% resistance to tetracycline, followed by lower levels of resistance to cefoxitin and imipenem (50%).

Lastly, two out of the twenty-three tested strains (9%) displayed a multidrug resistant (MDR) profile (Table 2).

##### Nădrag and Surduc Hunting Grounds (Timiș County)

In samples collected from the Nădrag and Surduc hunting grounds, the resistance phenotypes of nine *E. coli* strains isolated from red fox carcasses followed a trend similar to those observed in the previously mentioned groups. Susceptibility was high for trimethoprim–sulfamethoxazole (100%), followed by nitrofurantoin (89%) and cefoxitin, gentamicin, tobramycin, tetracycline, nalidixic acid, and chloramphenicol (67%). On the other hand, the tested isolates were more resistant to ceftazidime and amoxicillin (67%), followed by ampicillin, ciprofloxacin (56%), and cephalexin, imipenem, and amikacin (44%) (Table 2). It is noteworthy that three *E. coli* isolates showed resistance to between 12 and 14 of the antibiotics tested. In addition, three other isolates (33.4%) were classified as multidrug resistant (MDR).

Resistance to several molecules was noted for the *Proteus vulgaris* strain, including third-generation cephalosporins (ceftazidime) and first-generation cephalosporin (cephalexin), all aminoglycosides, and potentiated sulfonamides (Table 2).

##### Belinț-Chizătău, Paniova, and Valea Lungă Hunting Grounds (Timiș County)

Of the five bacterial strains isolated from foxes in these areas, only one *E. coli* strain and one *P. mirabilis* strain exhibited elevated resistance, both being classified as multidrug resistant (MDR) (Table 3). The other isolates demonstrated greater susceptibility to the antimicrobial drugs tested.

##### Silvaș and Zeicani Hunting Grounds (Hunedoara County)

In Hunedoara County, four bacterial strains were analyzed, consisting of *E. coli* (n = 3) and *Enterobacter* spp. (n = 1). All exhibited a low resistance profile relative to the tested antimicrobials, as shown in Table 3.

##### Crocna-Zimbru Hunting Ground (Arad County)

Finally, the *E. coli* strain isolated from this hunting ground tested as susceptible to 80% (12 out of 15) of the antimicrobial molecules, with resistance recorded only to ciprofloxacin, nalidixic acid, and chloramphenicol.

### 2.2. Antimicrobial Susceptibility Results for Some Gram-Positive Bacterial Species After the Disk Diffusion Method

Results regarding the antimicrobial susceptibility of Gram-positive bacterial strains were obtained after testing 64 isolates belonging to the genus *Staphylococcus* against 14 different antimicrobial agents.

#### 2.2.1. Peri-Urban Hunting Grounds

##### Buziaș and Moșnița Hunting Grounds (Timiș County) 

According to the results presented in Table 4, isolates of *S. xylosus* (n = 3) and *S. vitulinus* (n = 2) were fully susceptible to all tested agents. More broadly, all *Staphylococcus* strains (n = 20) were susceptible to chloramphenicol, while the majority (95%) showed susceptibility to cephalosporins (cefoxitin, cephalexin), imipenem, amikacin, and ciprofloxacin.

In contrast, resistance was most pronounced against oxacillin (50%), benzylpenicillin (45%), and tetracycline (40%). Overall, 10 isolates (50%) exhibited a multidrug-resistant (MDR) phenotype, being resistant to up to seven different antibiotic classes (Table 4). These included strains of *S. aureus*, *S. pseudintermedius*, *S. lentus*, *S. sciuri*, and *S. chromogenes*. Of particular interest was one *S. pseudintermedius* isolate, which displayed resistance to 86% (12 out of 14) of the six drugs tested, including cefoxitin, thereby qualifying as methicillin resistant [27]. The four *S. aureus* isolates expressed a higher resistance to tetracycline and erythromycin (3 out of 4); followed by gentamycin and trimethoprim-sulfamethoxazole (2 out of 4); and, to a lesser extent, to benzylpenicillin (1 out of 4).

##### Făget, Traian Vuia, and Margina Hunting Grounds (Timiș County)

The tested staphylococcal strains isolated from these hunting grounds showed increased responsiveness to the tested molecules, as can be seen in Table 4. Thus, all isolates were susceptible to cephalosporins (cefoxitin, cephalexin), imipenem, erythromycin, and chloramphenicol. One *S. vitulinus* strain was fully susceptible, while the remaining isolates were resistant to molecules of one (*S. vitulinus*), two (*S. equorum*), three (*S. sciuri*, n = 2*; S. vitulinus*), or four antibiotics (*S. sciuri*, n = 2) simultaneously.

#### 2.2.2. Peri-Rural Hunting Grounds

##### Oloșag, Boldur, and Sacoșul Mare Hunting Grounds (Timiș County)

The 24 *Staphylococcus* isolates examined from these hunting grounds displayed results comparable to those described in the Oloșag, Boldur and Sacoșul Mare hunting grounds (Table 5). The majority displayed high levels of susceptibility, with 95.8% sensitive to chloramphenicol and amikacin, 91.7% to cephalosporins (cefoxitin, cephalexin) and ciprofloxacin, and 87.5% to imipenem and trimethoprim–sulfamethoxazole. In addition, nearly half of the isolates (45.8%) were fully susceptible to all tested antimicrobial molecules.

The four *S. aureus* isolates expressed a high resistance to clindamycin (4/4) and tetracycline, benzylpenicillin, and gentamycin (3 out of 4); followed by imipenem, ciprofloxacin and trimethoprim-sulfamethoxazole (2 out of 4); and, to a lesser extent, to amikacin (1 out of 4).

Among the isolates, nine (37.5%) exhibited a multidrug-resistant (MDR) phenotype, resisting up to 11 different antibiotics. These MDR strains included the following species: *S. aureus* (n = 4), *S. pseudintermedius* (n = 1), *S. sciuri* (n = 2), *S. lentus* (n = 1), and *S. xylosus* (n = 1).

##### Nădrag and Surduc Hunting Grounds (Timiș County)

A total of six *Staphylococcus* isolates were examined from these hunting grounds (Table 5). All strains exhibited resistance to at least two of the tested antimicrobial agents, the most susceptible being *S. lentus*, which remained sensitive to 86% of the antibiotics. The remaining five isolates were each resistant to at least four drugs, with up to seven in the case of *S. pseudintermedius* (50% resistance), which was also identified as methicillin resistant.

On the other hand, all isolates were susceptible to amikacin and chloramphenicol, while 83% exhibited susceptibility to cephalosporins (cefoxitin, cephalexin), imipenem, and erythromycin. Oxacillin and penicillin G demonstrated relatively higher efficacy (66.7%), although notable resistance was observed to clindamycin (83%), ciprofloxacin (66.7%), tetracycline (66.7%), trimethoprim–sulfamethoxazole (50%), gentamicin (50%), and tobramycin (50%). Furthermore, four of the six isolates (66.7%) exhibited a phenotypic multidrug-resistant (MDR) profile.

##### Belinț-Chizătău Hunting Ground (Timiș County)

A single strain of *S. vitulinus* was tested from this site; it showed resistance to every antibiotic except erythromycin and chloramphenicol, moreover it was classified as methicillin resistant due to cefoxitin resistance and MDR (Table 6).

##### Silvaș and Zeicani Hunting Grounds (Hunedoara County)

There was less resistance in these three tested species. Thus, *S. vitulinus* was resistant to tobramycin, *S. sciuri* was resistant to oxacillin and benzylpenicillin, and *S. xylosus* was susceptible to every antibiotic that was tested (Table 6).

##### Crocna-Zimbru Hunting Ground (Arad County)

Finally, the two strains of *S. lentus* and *S. xylosus* isolated from foxes in this area tested as resistant only to oxacillin and benzylpenicillin.

### 2.3. Antimicrobial Susceptibility Results Determined with the Vitek 2 Compact for Some Gram-Negative Bacterial Species

The Vitek 2 Compact system was used to analyze a subset of isolates in order to look more closely at phenotypic antimicrobial resistance in bacteria linked to wildlife. From the 137 strains, a subset was chosen for Vitek 2 testing based on initial MDR classification, resistance to two antibiotic classes, or scarcity of published data. Therefore, the tested bacteria included 41 Gram-negative strains, belonging to the species *E. coli* (n = 26), *P. mirabilis* (n = 10), *P. vulgaris* (n = 1), *S. sonnei* (n = 2) and *S. enterica* subsp. *enterica* (n = 2). In addition, 27 Gram-positive strains of the genus *Staphylococcus* were examined, comprising the following species: *S. aureus* (n = 6), *S. pseudintermedius* (n = 6), *S. sciuri* (n = 6), *S. vitulinus* (n = 2), *S. xylosus* (n = 1), *S. lentus* (n = 3), *S. chromogenes* (n = 1) and *S. felis* (n = 2).

#### 2.3.1. Peri-Urban Hunting Grounds

##### Buziaș and Moșnița Hunting Grounds

A total of 13 bacterial strains were analyzed from foxes in the Buziaș and Moșnița hunting grounds. These included *E. coli* (n = 8), *P. mirabilis* (n = 3), and *S. enterica* subsp. *enterica* (n = 2).

The overall pattern of the *E. coli* isolates showed a higher resistance to beta-lactams and aminoglycosides, while all isolates were fully susceptible to nitrofurantoin and trimethoprim–sulfamethoxazole, and in high rates (87.5%), susceptible to cephalexin and tetracycline. Therefore, seven strains (87.5%) were resistant to amikacin, five strains (62.5%) showed resistance to ampicillin, and four strains (50%) were resistant to amoxicillin/clavulanic acid and imipenem (Table 7).

Regarding the *P. mirabilis* isolates, one strain expressed resistance to cephalexin, cefpodoxime, and gentamicin. The two *S. enterica* strains displayed significant resistance to six (54.55%) and seven (63.63%) antimicrobial molecules, respectively. These included ampicillin, amoxicillin–clavulanic acid, gentamicin, imipenem, tetracycline, and trimethoprim–sulfamethoxazole, and additionally, cephalexin, cefpodoxime, amikacin, and chloramphenicol (Table 7).

##### Făget, Traian Vuia, and Margina Hunting Grounds

From these hunting grounds, seven *E. coli* strains were analyzed using the Vitek 2 Compact system. As shown in Table 7, six of the seven isolates (86%) were highly susceptible to the tested antimicrobial agents. Complete susceptibility (100%) was observed to trimethoprim–sulfamethoxazole, nitrofurantoin, and chloramphenicol, followed by a lesser value for cefpodoxime (75%). Moderate resistance rates were detected for amikacin (57%) and gentamicin (43%). In contrast, one *E. coli* isolate exhibited a more pronounced resistance profile, including ampicillin, amoxicillin–clavulanic acid, cefpodoxime, imipenem, gentamicin, and amikacin.

#### 2.3.2. Peri-Rural Hunting Grounds

##### Oloșag, Boldur, and Sacoșul Mare Hunting Grounds

Out of 23 Gram-negative isolates previously tested by disk diffusion, fourteen strains were selected for Vitek 2 testing. These included the species *E. coli* (n = 6), *P. mirabilis* (n = 6), and *Shigella sonnei* (n = 2) (Table 8).

Among the *E. coli* strains, the majority were resistant to ampicillin (83%), followed by imipenem (67%), and amoxicillin/clavulanic acid and amikacin (50%). Moreover, the observed susceptibility patterns were consistent with those previously reported for isolates from the Buziaș and Moșnița hunting grounds, including 100% susceptibility to trimethoprim–sulfamethoxazole and 83% susceptibility to cefpodoxime, tetracycline, nitrofurantoin, and chloramphenicol.

All six *P. mirabilis* isolates showed a generally favorable antimicrobial susceptibility profile, remaining susceptible to the majority of the tested agents. Nevertheless, resistance to trimethoprim–sulfamethoxazole was detected in two isolates, while resistance to cephalexin was observed in one isolate. Similarly, *S. sonnei* isolates demonstrated a high overall level of susceptibility to the antimicrobials evaluated, with resistance detected only sporadically, namely, to tetracycline and imipenem, each in a single isolate (Table 8).

##### Nădrag and Surduc Hunting Grounds

In this subset, antimicrobial susceptibility testing was conducted on four *E. coli* isolates and one *P. vulgaris* isolate. The *E. coli* isolates displayed high levels of resistance to several commonly used antimicrobials, including ampicillin, amoxicillin–clavulanic acid, cephalexin, imipenem, amikacin, and gentamicin, with resistance observed in 75% of the isolates. In contrast, complete susceptibility (100%) was recorded for trimethoprim–sulfamethoxazole and nitrofurantoin, while a high susceptibility rate (75%) was observed for cefpodoxime. Notably, two *E. coli* isolates shared identical multidrug-resistant (MDR) profiles, characterized by concurrent resistance to multiple antimicrobial classes, namely β-lactams (ampicillin, amoxicillin–clavulanic acid, and cephalexin), aminoglycosides (amikacin and gentamicin), imipenem, tetracycline, and chloramphenicol (Table 8).

Furthermore, the *P. vulgaris* isolate exhibited resistance to five of the tested antimicrobial agents, including cephalosporins (cephalexin and cefpodoxime), aminoglycosides (amikacin and gentamicin), and trimethoprim–sulfamethoxazole (Table 8).

##### Belinț-Chizătău and Paniova Hunting Ground

From the red fox samples collected in these hunting grounds, one isolate of *E. coli* and one of *P. mirabilis* were selected for analysis with the Vitek 2 Compact. The *E. coli* strain was resistant to five antibiotics, including ampicillin, amoxicillin-clavulanic acid, cephalexin, amikacin, and gentamicin, while the *P. mirabilis* strain showed resistance to four antibiotics, namely, cephalexin, cefpodoxime, amikacin, and gentamicin.

### 2.4. Antimicrobial Susceptibility Results for Some Gram-Positive Bacterial Species with the Vitek 2 Compact

In the examination with the Vitek 2 Compact system, 27 *Staphylococcus* strains previously identified as multidrug-resistant (MDR) by the disk diffusion method were tested. These isolates represented eight species: *S. aureus* (n = 6), *S. pseudintermedius* (n = 6), *S. sciuri* (n = 6), *S. vitulinus* (n = 2), *S. xylosus* (n = 1), *S. lentus* (n = 3), *S. chromogenes* (n = 1), and *S. felis* (n = 2).

#### 2.4.1. Peri-Urban Hunting Grounds

##### Buziaș and Moșnița Hunting Grounds

Among the multidrug-resistant (MDR) isolates obtained from foxes in the Buziaș and Moșnița hunting grounds, ten *Staphylococcus* strains were analyzed with the Vitek 2 system. Cefoxitin screening identified methicillin resistance in two *S. pseudintermedius* strains and one *S. sciuri* strain, all considered resistant to beta-lactams. These isolates also showed resistance to clindamycin, with one *S. pseudintermedius* strain resistant to 10 (71%) out of the 14 tested molecules (Table 9).

As for the *S. aureus* isolates, in addition to being fully resistant (100%) to tetracycline, erythromycin, and trimethoprim–sulfamethoxazole, half of them also exhibited resistance to benzylpenicillin and gentamicin. In contrast, notable susceptibility was recorded to chloramphenicol (100% of the staphylococci) and kanamycin, enrofloxacin, and marbofloxacin (90%), as well as oxacillin, cephalothin, gentamicin, and erythromycin (70%) (Table 9).

##### Traian Vuia Hunting Ground

The single tested *S. sciuri* strain isolated from a male red fox in the Traian Vuia hunting ground showed resistance to benzylpenicillin, amoxicillin–clavulanic acid, and clindamycin.

#### 2.4.2. Peri-Rural Hunting Grounds

##### Oloșag, Boldur, and Sacoșul Mare Hunting Grounds

From these hunting grounds, ten staphylococcal strains were further analyzed. Vitek 2 testing revealed a striking methicillin resistance in 60% of the isolates, which was confirmed by cefoxitin screening. This phenotype was detected across multiple species, including *S. aureus* (3/4 strains), *S. pseudintermedius* (1/2 strains), and *S. sciuri* (2/2 strains) (Table 10).

Compared with *S. aureus* isolates from Buziaș and Moșnița, these strains exhibited a broader resistance profile. High resistance rates were observed to beta-lactams, aminoglycosides (gentamicin 75%, kanamycin 50%), fluoroquinolones (enrofloxacin 50%, marbofloxacin 50%), tetracycline (75%), clindamycin (75%), and trimethoprim–sulfamethoxazole (50%). All isolates were resistant to benzylpenicillin but remained fully susceptible to chloramphenicol (Table 10).

##### Nădrag and Surduc Hunting Grounds

From the Nădrag and Surduc hunting grounds, five *Staphylococcus* isolates were examined, including four species: *S. pseudintermedius*, *S. sciuri*, *S. felis*, and *S. vitulinus*. Similar to the results from the Oloșag, Boldur, and Sacoșul Mare hunting grounds, *S. pseudintermedius* and *S. sciuri* from Nădrag and Surduc exhibited the methicillin-resistance phenotype. Moreover, these strains were fully susceptible to chloramphenicol and kanamycin (100%) and showed only minimal resistance to erythromycin (20%). In contrast, *S. felis* and *S. vitulinus* isolates were susceptible to beta-lactams but exhibited high resistance to clindamycin (80%) and trimethoprim–sulfamethoxazole (60%) (Table 10).

##### Belinț-Chizătău Hunting Ground

A single *S. vitulinus* isolate from the Belinț-Chizătău hunting ground was examined, and tested as resistant to 71% of the antimicrobial drugs, including methicillin; however, it remained susceptible to marbofloxacin, erythromycin, and chloramphenicol.

### 2.5. Data Analysis and Interpretation

McNemar’s test was applied to assess the agreement between the disk diffusion (DD) and Vitek 2 methods for antimicrobial susceptibility testing of both Gram-negative and Gram-positive strains, considering only antibiotics common to both systems. For Gram-negative isolates, a total of 41 strains were tested by both methods, with some exceptions for specific antibiotics as indicated in Table 11 (column 2). Disk diffusion included 15 antibiotics from seven antimicrobial classes, while Vitek 2 included 11 antibiotics from six classes; therefore, the comparison was limited to shared agents, with the exception of ceftazidime (DD) and cefpodoxime (Vitek 2), which were grouped as third-generation cephalosporins. Overall, no statistically significant differences were observed between the two methods for most antibiotics, indicating a high level of concordance. A significant difference was detected only for the third-generation cephalosporins group, which showed a higher number of discordant results (*p* = 0.001).

For Gram-positive isolates, 27 strains were tested by both methods. Disk diffusion included 15 antibiotics from eight antimicrobial classes, while Vitek 2 included 14 antibiotics from the same eight classes; thus, only shared antibiotics were analyzed. No statistically significant differences were observed between DD and Vitek 2 for any of the antibiotics tested, with all McNemar *p*-values exceeding 0.05, as shown in Table 12. Although oxacillin and cefoxitin exhibited relatively higher discordance, these differences did not reach statistical significance. For chloramphenicol, all isolates were classified as susceptible by both methods, resulting in no discordant pairs and rendering McNemar’s test not applicable. Taken together, these results demonstrate overall good agreement between DD and Vitek 2 for susceptibility testing of both Gram-negative and Gram-positive bacteria.

Taken together, these findings demonstrate an overall high level of agreement between disk diffusion and Vitek 2 for antimicrobial susceptibility testing of both Gram-negative and Gram-positive isolates. Based on this concordance, disk diffusion results were retained for subsequent analyses, as this method encompassed a broader range of antimicrobial agents and allowed a more comprehensive characterization of resistance patterns in the studied isolates. Additionally, imipenem susceptibility was supported by MIC-based testing, strengthening the reliability of carbapenem resistance assessments in this dataset.

The chi-square test for independence was employed to evaluate potential differences in the antimicrobial susceptibility profiles of Gram-positive and Gram-negative bacterial strains isolated from peri-urban and peri-rural areas using the disk diffusion method. A total of 64 Gram-positive and 73 Gram-negative isolates were included in the analysis (Table 13 and Table 14). For Gram-negative isolates, strains exhibiting intrinsic resistance were excluded from the corresponding analyses, as indicated in the table legends.

Across all antibiotics tested, no statistically significant association was observed between the area of isolation and the antimicrobial resistance or susceptibility patterns for either Gram-positive or Gram-negative strains, with all *p*-values exceeding the threshold for statistical significance (*p* > 0.05).

**Figure 1:**
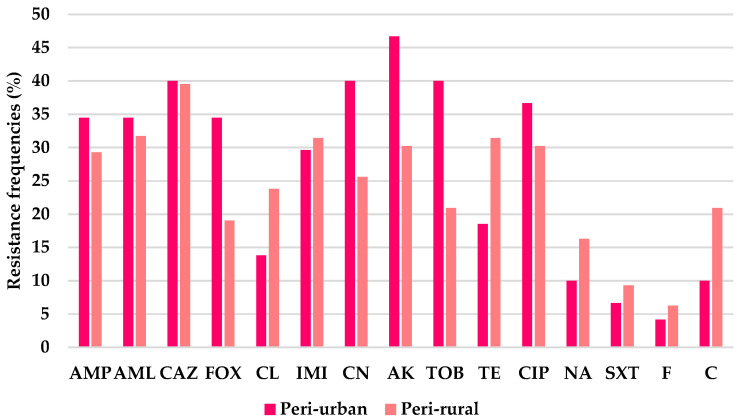
Overall distribution of resistance trends in Gram-negative isolates from peri-urban and peri-rural hunting grounds.

However, variations in resistance and susceptibility frequencies were noted between peri-urban and peri-rural isolates for several antibiotics in both groups. In the Gram-negative group, resistance was highest to ceftazidime (CAZ, 39.7%), followed by amikacin (AK, 37.0%), then ciprofloxacin (CIP, 32.9%) and amoxicillin (AML, 32.9%). Similar resistance levels were observed for gentamicin (CN, 31.5%), ampicillin (AMP, 31.4%), and imipenem (IMI, 30.6%), followed by tobramycin (TOB, 28.8%). Moderate resistance was detected for tetracycline (TE, 25.8%), cefoxitin (FOX, 25.4%), and cephalexin (CL, 19.7%). Lower resistance frequencies were recorded for chloramphenicol (C, 16.4%), nalidixic acid (NA, 13.7%), trimethoprim–sulfamethoxazole (SXT, 8.2%), and nitrofurantoin (F, 5.4%). Resistance frequencies tended to be higher in peri-urban isolates for eight of the antibiotics, and particularly for cefoxitin (34.5% in peri-urban versus 19% in peri-rural), gentamicin (40% vs. 25.6%), amikacin (46.7% vs. 30%), and tobramycin (40% vs. 21%). In contrast, peri-rural isolates showed higher resistance rates to cephalexin (24% in peri-rural versus 14% in peri-urban), tetracycline (31.5% vs. 18.5%), and chloramphenicol (21% vs. 10%). Low resistance levels (<10%) were observed in both settings for trimethoprim–sulfamethoxazole and nitrofurantoin (Figure 1).

In the Gram-positive group, resistance was highest to penicillin (P, 46.9%) and oxacillin (OX, 46.4%), followed by tetracycline (TE, 35.9%) and clindamycin (CD, 35.9%), and then tobramycin (TOB, 31.3%). Moderate resistance levels were observed for gentamicin (CN, 26.6%) and trimethoprim–sulfamethoxazole (SXT, 25.0%), while lower resistance was detected for ciprofloxacin (CIP, 14.1%), erythromycin (E, 10.9%), and imipenem (IMI, 9.4%). Very low resistance frequencies were recorded for cefoxitin (FOX, 7.8%), cephalexin (CL, 7.8%), amikacin (AK, 6.3%), and chloramphenicol (C, 1.6%).

**Figure 2:**
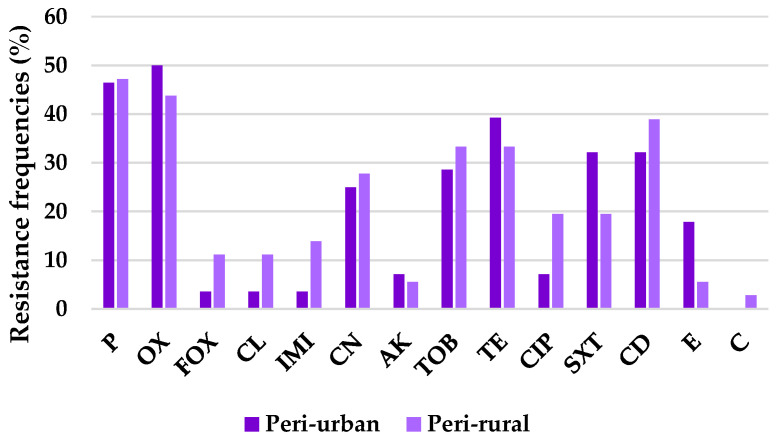
Overall distribution of resistance trends in Gram-positive isolates from peri-urban and peri-rural hunting grounds.

Interestingly, resistance frequencies tended to be higher in peri-rural isolates for nine out of the fourteen antibiotics, specifically, cefoxitin and cephalexin (11% in peri-rural versus 3.6% in peri-urban), imipenem (14% vs. 3.6%), ciprofloxacin (19.5% vs. 7%), and chloramphenicol (3% vs. 0%). In contrast, peri-urban isolates expressed increased rates for trimethoprim-sulfamethoxazole (32% vs. 19.5%), and erythromycin (18% vs. 5.6%) (Figure 2).

Despite the descriptive observations, the statistical findings demonstrate comparable antimicrobial susceptibility profiles among both Gram-positive and Gram-negative bacterial isolates from the peri-urban and peri-rural settings, indicating that geographic origin did not significantly influence resistance patterns within the study population.

**Figure 3:**
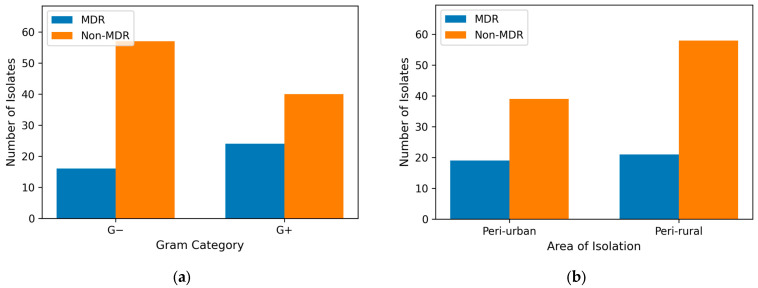
(**a**) Distribution of MDR and non-MDR isolates by peri-urban and peri-rural areas; (**b**) Distribution of MDR and non-MDR isolates among Gram-negative and Gram-positive bacteria.

Despite the slightly increased prevalence of MDR strains in peri-urban (32.76%) compared to peri-rural hunting grounds (26.59%), there was no statistically significant association between area of isolation and MDR status (*p* > 0.05). This indicates that the distribution of MDR and non-MDR isolates was comparable between peri-urban and peri-rural settings (Figure 3a). Given the absence of a spatial effect, subsequent analyses focused on biological factors that might better explain variability in multidrug resistance. Gram-positive isolates exhibited a higher proportion of multidrug resistance (24/64), compared to Gram-negative isolates (16/73) (χ^2^ = 4.24, df = 1, *p* < 0.05) (Figure 3b). Conversely, non-MDR isolates were more frequent among Gram-negative bacteria. These findings suggest that Gram-positive bacteria may contribute disproportionately to the burden of multidrug resistance in the studied population.

Methicillin resistance in staphylococcal isolates was assessed using MIC values obtained via the Vitek 2 system. This approach was chosen due to the diversity of species studied and the greater accuracy of MIC in detecting methicillin resistance, in accordance with EUCAST guidelines. Methicillin-resistant staphylococci (MRS) were more frequently detected in foxes from peri-rural areas (9/16; 56.3%) than in those from peri-urban areas (3/11; 27.3%).

## 3. Discussion

The present study investigated phenotypic AMR in bacterial isolates obtained between 2022 and 2024 from red foxes (n = 108) across sixteen hunting grounds in western Romania. Samples were collected post mortem from animals that had died due to hunting-related gunshot wounds [28,29]. The aim was to evaluate the role of red foxes as potential sentinels for monitoring AMR dynamics in ecosystems shaped by interactions between humans, domestic animals, and wildlife.

The red fox (*Vulpes vulpes*) has been increasingly recognized as a valuable One Health sentinel for AMR surveillance because of its wide geographic distribution, opportunistic feeding habits, and adaptability to both rural and urban environments [30]. By exploiting diverse food sources, foxes are regularly exposed to microbial reservoirs such as small mammals and birds, as well as to anthropogenic contamination originating from human activity, agriculture, and pharmaceutical waste [31,32]. This ecological positioning at the interface of natural and human-modified habitats makes them particularly suitable for tracking the circulation of AMR across wildlife, domestic animals, and humans [33,34,35,36].

Interpretation of antimicrobial resistance prevalence was based on resistant (R) isolates only, with “Susceptible, Increased Exposure” (I) categories treated as susceptible, in accordance with current EUCAST definitions. This approach avoids overestimation of resistance at the population level, which is particularly relevant in wildlife surveillance studies, in which antimicrobial exposure is indirect.

Out of the 137 strains, a subset of isolates initially tested by Kirby–Bauer Disk-Diffusion was selected for Vitek 2 analysis. This subset included isolates initially classified as MDR, i.e., those resistant to at least two antibiotic classes, as well as isolates representing species for which limited data exist in the wildlife studies (e.g., *Proteus* spp., *Shigella sonnei*).

The comparison between disk diffusion (DD) and the Vitek 2 system revealed an overall high level of agreement for antimicrobial susceptibility testing in both Gram-negative and Gram-positive isolates, supporting the reliability of DD as a routine screening method. For Gram-negative bacteria, the absence of statistically significant differences for most antibiotics suggests a strong concordance between the two methods, which is consistent with previous reports showing good categorical agreement between disk diffusion and MIC-based automated systems for many drug–organism combinations [37,38]. The significant discordance observed for third-generation cephalosporins, however, highlights a known limitation of disk diffusion, as beta-lactam antibiotics, particularly cephalosporins, are more prone to methodological discrepancies due to borderline MIC values and complex resistance mechanisms such as ESBL production [37,39]. This finding underscores the importance of confirmatory MIC-based testing when resistance to critical antibiotic classes is suspected.

For Gram-positive isolates, complete concordance between DD and Vitek 2 across all tested antibiotics further supports the robustness of disk diffusion for these organisms. Although oxacillin and cefoxitin showed relatively higher discordance, the lack of statistical significance suggests that these differences were limited and unlikely to affect overall clinical interpretation. The perfect agreement observed for chloramphenicol reinforces the consistency of susceptibility categorization for antibiotics with well-defined breakpoints. Taken together, these results align with works in the existing literature indicating that while disk diffusion generally provides results comparable to MIC-based methods, discrepancies may arise for specific antibiotics or resistance phenotypes, emphasizing the complementary role of automated systems in antimicrobial resistance surveillance and clinical decision-making [37].

Although no statistically significant association was observed between area of isolation and antimicrobial susceptibility for either Gram-positive or Gram-negative isolates, descriptive differences in resistance frequencies were evident between peri-urban and peri-rural settings. These trends, such as higher resistance to aminoglycosides and cefoxitin among peri-urban Gram-negative isolates and increased resistance to several antibiotics (cefoxitin, cefalexin, imipenem, and ciprofloxacin) among peri-rural Gram-positive strains, may reflect local antibiotic usage practices or environmental exposures that were not sufficient to produce statistically detectable differences [32]. Additionally, the lack of consistent differences between peri-urban and peri-rural isolates could indicate shared environmental exposures. Given that the investigated hunting grounds are connected by common river systems and water sources, such connectivity may contribute to similarities in bacterial communities and resistance profiles [32,40]; however, this interpretation remains speculative in the absence of environmental sampling.

Both peri-urban and peri-rural hunting grounds, which are more anthropogenically influenced (e.g., Buziaș, Moșnița, Oloșag, Boldur, Sacoșul Mare, Nădrag, Surduc, Făget, Traian Vuia, and Margina), harbored bacteria with a broader diversity of antimicrobial resistance (AMR) phenotypes and a higher frequency of multidrug resistance (MDR). In these locations, *Escherichia coli* isolates commonly displayed resistance to beta-lactams, including third-generation cephalosporins such as ceftazidime, as well as to aminoglycosides, and, in certain groups, carbapenems (imipenem). Fluoroquinolone resistance was also sporadically detected. Methicillin-resistant staphylococci were also identified in samples from the same hunting grounds. By contrast, isolates from forest-dominated areas with limited human or agricultural pressure (e.g., Silvaș, Zeicani, and Crocna-Zimbru) generally retained high susceptibility to chloramphenicol, nitrofurantoin, and trimethoprim–sulfamethoxazole.

Additionally, the lack of consistent differences between peri-urban and peri-rural isolates may reflect shared environmental exposures, as the investigated hunting grounds are connected by common river systems and water sources, which could contribute to a homogenization of bacterial communities and antimicrobial resistance determinants [30,37].

Comparable trends have been reported in other European countries. Studies from Denmark, Poland, Norway, the United Kingdom, and Portugal demonstrated that foxes living near rural settlements or livestock facilities carried higher AMR rates and frequent MDR profiles, including methicillin-resistant staphylococci and *E. coli* resistant to beta-lactams (e.g., cefotaxime, ampicillin) and tetracycline, whereas isolates from remote forested habitats remained largely susceptible [7,11,23,41,42,43]. Together, these findings support the hypothesis that red foxes act as ecological bridges facilitating the transfer and maintenance of antimicrobial resistance between anthropogenically influenced environments and natural ecosystems [44,45].

Within the Romanian dataset, Gram-negative bacteria expressed a higher overall resistance to ceftazidime, followed by amikacin, ciprofloxacin, amoxicillin, gentamicin, ampicillin, and imipenem, with particularly elevated resistance observed in *E. coli* and *Salmonella* isolates. Resistance in *E. coli* was frequent for ceftazidime (up to 67%), ciprofloxacin (up to 56%), imipenem (up to 44%), tetracycline (up to 33%), gentamicin (up to 40%), and ampicillin (up to 56%), whereas trimethoprim–sulfamethoxazole, nitrofurantoin, and chloramphenicol remained mostly effective (67–100%). Similar high-risk profiles have been documented in Poland, Portugal, Ireland and Norway, where foxes carried extended-spectrum beta-lactamase (ESBL)-producing *E. coli* [7,41,42,43,46,47].

At the species level, *E. coli* isolates showed consistently elevated resistance to ceftazidime, with resistance rates ranging from approximately 40–50% in peri-rural hunting grounds to up to 50% in peri-urban areas, while susceptibility to trimethoprim–sulfamethoxazole and nitrofurantoin remained uniformly high (89–100%) across all sites. Furthermore, in the peri-rural Nădrag and Surduc hunting grounds, *E. coli* isolates displayed the highest resistance levels recorded in this study, with resistance rates of 67% to ceftazidime and amoxicillin, 56% to ampicillin and ciprofloxacin, and 44% to cephalexin, imipenem, and amikacin. The Surduc area encompasses an artificial lake that supports recreational activities, seasonal tourism, and the presence of campgrounds, vacation homes, and rental properties, with direct discharge of untreated wastewater into the lake, all of which may contribute to elevated anthropogenic pressure on the local environment. Although no environmental samples were analyzed and no direct assessment of water contamination was performed, previous studies have shown that waste waters, and even natural waters, can act as reservoirs for antimicrobial-resistant bacteria [7,48,49,50]. This contrasting resistance–susceptibility pattern has been frequently reported in wildlife-associated *E. coli* and reflects phenotypic adaptation to beta-lactam selective pressure, without implying confirmed ESBL production in the absence of molecular evidence. In *Proteus* spp. from red foxes, multidrug-resistant profiles were observed. However, resistance to imipenem, tetracycline, and nitrofurantoin reflects intrinsic species traits and was excluded from the calculations. Importantly, several isolates showed additional acquired resistance, highlighting the epidemiological role of foxes as carriers of resistant *Proteus* species. This is significant as *Proteus* can act as a genetic hub in the gut microbiome, transferring resistance genes via mobile elements to other enterobacteria, thereby amplifying and maintaining a reservoir of AMR within wildlife populations and the environments they traverse [7,42,51,52,53]. Although less frequent, *Shigella sonnei* and *Salmonella enterica* isolates revealed localized resistance patterns. Among the *S. sonnei* strains from Oloșag, Boldur, and Sacoșul Mare, all isolates were resistant to tetracycline, while partial resistance was observed for cefoxitin and imipenem. *S. enterica* isolates remained susceptible to cephalexin, despite resistance to several other beta-lactams, aminoglycosides, and tetracycline. Similar observations in other European studies suggested that these localized resistance patterns could reflect environmental contamination pressures rather than species-wide adaptations [7,42,47].

The staphylococcal isolates comprising the Gram-positive group exhibited an overall favorable antimicrobial susceptibility profile, with chloramphenicol and amikacin remaining highly effective, followed by cephalosporins and imipenem. In contrast, resistance was predominantly observed against beta-lactams (penicillin, oxacillin), tetracycline, clindamycin, and selected aminoglycosides. Methicillin resistance was identified in *S. aureus, S. pseudintermedius* and *S. sciuri*, paralleling findings from the United Kingdom, Ireland, and Poland, where coagulase-negative staphylococci (*S. aureus, S. sciuri*, *S. equorum*, *S. capitis*) from foxes frequently contained the *mecA* gene, with prevalence reaching 89% [3,54].

At the species level, *Staphylococcus aureus* exhibited the most consistent and elevated resistance among Gram-positive isolates, particularly to tetracycline (75%), gentamicin (62.5%), benzylpenicillin (50%), clindamycin (50%), and trimethoprim–sulfamethoxazole (50%), while resistance to ciprofloxacin and imipenem remained lower (25%), and susceptibility to amikacin and chloramphenicol was largely preserved. In contrast, other staphylococcal species demonstrated more heterogeneous and often narrower resistance profiles. *S. xylosus* and *S. vitulinus* frequently remained fully susceptible or resistant to only a limited number of antibiotics, whereas *S. sciuri*, *S. lentus*, and *S. pseudintermedius* showed broader resistance patterns, most commonly involving beta-lactams, tetracycline, and clindamycin. Notably, resistance to oxacillin and benzylpenicillin was recurrent across multiple non-aureus species, while susceptibility to chloramphenicol and amikacin was consistently maintained. This species-dependent variability highlights the ecological complexity of staphylococcal resistance in wild red foxes and suggests that, alongside *S. aureus*, coagulase-negative staphylococci may serve as important reservoirs of selectively maintained resistance traits in wildlife-associated bacterial communities [3,20,54,55]. Notably, the highest resistance levels among Gram-positive isolates were recorded in the Nădrag and Surduc hunting grounds, where resistance was observed for clindamycin (83%), ciprofloxacin, tetracycline, and oxacillin (each 66.7%), despite complete susceptibility to chloramphenicol and amikacin. Similar to the Gram-negative findings, the Surduc area is characterized by increased human activity linked to recreational use, which may contribute to elevated environmental selective pressure. Although no environmental matrices were analyzed and causal inference cannot be established, previous studies have identified anthropogenically influenced waters as potential reservoirs for resistant staphylococci [7,48,49,50].

Methicillin resistance (MR) in staphylococcal isolates was assessed using the MIC values obtained via the Vitek 2 system. This approach was chosen due to the diversity of species studied and the greater accuracy of MIC in detecting methicillin resistance, in accordance with standard guidelines [26].

Methicillin-resistant staphylococci (MRS) were more frequently detected in foxes from peri-rural areas (9/16; 56.3%) than from peri-urban areas (3/11; 27.3%). Notably, three methicillin-resistant *Staphylococcus aureus* (MRSA) isolates were identified exclusively in peri-rural hunting grounds, underscoring the public health relevance of these findings, as MRSA remains one of the most important zoonotic and community-associated antimicrobial-resistant pathogens worldwide [56,57]. The higher prevalence of methicillin-resistant staphylococci observed in foxes from peri-rural areas compared with peri-urban environments may reflect differences in ecological exposure to antimicrobial resistance reservoirs [58,59]. Peri-rural habitats are closely associated with livestock farming, where the therapeutic and metaphylactic use of antimicrobials, particularly beta-lactams, exerts selective pressure favoring methicillin-resistant strains [60]. Livestock-associated staphylococci, including both MRSA and methicillin-resistant coagulase-negative staphylococci, can disseminate into the environment through manure, slurry application, and contaminated soils and water sources [61]. Foxes inhabiting these areas frequently interact with the wildlife–livestock interface through predation, scavenging of animal remains, and contact with farm environments, increasing their likelihood of acquiring resistant bacteria [62]. In addition, rural ecosystems may promote the persistence and horizontal transfer of resistance determinants such as *mec*A and *mec*C via mobile genetic elements, supported by high bacterial diversity and environmental co-localization. Notably, *mec*C-positive staphylococci have been repeatedly associated with ruminants and wildlife species in rural settings, suggesting that peri-rural environments may act as important reservoirs for these lineages [63]. In contrast, other studies pointed out that peri-urban foxes are more likely exposed to human-associated staphylococci, where environmental dissemination of resistant strains may be more limited, due to regulated waste management systems. Differences in diet and foraging behavior between peri-rural and peri-urban foxes may further contribute to the observed patterns [3,20].

In addition to MRSA, methicillin resistance was observed in other staphylococcal species with known veterinary and opportunistic human relevance. *Staphylococcus pseudintermedius*, a common pathogen in dogs and cats and a leading cause of skin and soft tissue infections, was detected among fox isolates, raising concerns regarding potential bidirectional transmission between wildlife and domestic animals, particularly in peri-urban and peri-rural interfaces where spatial overlap is likely [55,58]. Furthermore, *Staphylococcus sciuri* and other coagulase-negative staphylococci (CoNS), although often regarded as commensals, are increasingly implicated in infections in immunocompromised individuals and in patients with implanted medical devices [20]. The presence of methicillin resistance in these species provides an early warning signal of the presence of resistance at interfaces where humans, domestic animals, and wildlife converge, rather than evidence of active transmission pathways. This concern is reinforced by molecular evidence from the scientific literature, demonstrating that *mec*A and *mec*C genes detected in wildlife-associated staphylococci are identical or closely related to those circulating in human and veterinary clinical settings [57]. Overall, these findings reinforce a One Health perspective in which peri-rural wildlife, particularly red foxes, function as effective sentinels for the occurrence of antimicrobial resistance in environments influenced by human activity. Due to their ecological adaptability, occasional extra-territorial movements, and frequent use of landscapes at the interface of anthropogenic and natural habitats, red foxes are well positioned to reflect resistance pressures driven by agricultural and human activities [3,20].

Comparable observations and results have been reported in other European countries, including Ireland, Poland, and Norway, where methicillin-resistant *S. pseudintermedius* and CoNS were recovered from foxes in proximity to human settlements and livestock. In the United Kingdom, an investigation of 38 foxes revealed methicillin-resistant *S. sciuri* (35%), *S. equorum* (27%), and *S. capitis* (22%), with the *mec*A gene detected in 89% of isolates and broad beta-lactam resistance in 27% [3,45]. Although prevalence in wildlife is generally lower than in domestic animals, these findings collectively emphasize that foxes can harbor clinically significant staphylococci, reinforcing the need for integrated surveillance that encompasses wildlife, companion animals, and humans [64].

Despite the fact that a slightly higher prevalence of multidrug-resistant (MDR) strains was observed in peri-urban compared with peri-rural settings, this difference was not statistically significant, indicating that geographic origin alone did not substantially influence MDR distribution in the study population. This suggests that the emergence of MDR is likely driven by factors other than spatial location, such as shared selective pressures or the intrinsic bacterial characteristics present in each environment [58]. At the organism level, peri-urban isolates showed higher MDR proportions, with 23.3% of *Escherichia coli* and both *Salmonella enterica* isolates classified as MDR, compared with peri-rural settings, in which 14.6% of *E. coli* isolates and a single *Proteus vulgaris* isolate were MDR. Notably, the higher proportion of MDR among Gram-positive isolates points to a biological influence on resistance, suggesting that Gram-positive bacteria may play a larger role in the overall prevalence of multidrug resistance, regardless of location [60]. As an observation, isolates from peri-rural, forest rich areas, such as Silvaș/Zeicani and Crocna-Zimbru, displayed no MDR isolates, with *E. coli* found to be largely susceptible and staphylococci showing no evidence of methicillin resistance.

Previous studies in Europe have reported associations between human population density and MDR prevalence. In Norway, high MDR rates occurred in areas of intermediate-to-high human density, particularly in *E. coli* resistant to cefotaxime, ampicillin, and tetracycline [41]. In other research works, it has been noticed that foxes from peri-urban areas or livestock-associated landscapes carried frequently MDR *Enterobacteriaceae* and staphylococci, whereas those from remote forest habitats primarily yielded susceptible strains [7,42,43]. In Denmark, carbapenem- or colistin-resistant *E. coli* were detected in 387 of 528 fox samples, with higher prevalence in regions more densely populated by humans [11]. Taken together, these findings suggest that MDR clustering in red foxes may be influenced by anthropogenic pressures and that patterns documented in Romania are consistent with broader European trends [32,64].

In addition to the overall AMR and MDR patterns observed, methicillin-resistant staphylococci (MRS) represent a clinically relevant finding within the red fox populations studied. Staphylococci are important because of their role as common pathogens in companion animals and opportunistic agents in humans [36]. The identification of cefoxitin-positive isolates in foxes therefore provides a useful perspective for understanding the potential links between wildlife, domestic animals, and clinical settings. MRS were detected among free-living red foxes originating from both, peri-urban and peri-rural hunting grounds, with *S. aureus*, *S. pseudintermedius* and *S. sciuri* representing the main cefoxitin-positive species.

The findings of this study can be understood within a One Health framework, emphasizing the interconnectedness of human, animal, and environmental health, and highlighting how antimicrobial resistance (AMR) exemplifies this interdependence [65]. The red fox, with its global distribution, ecological adaptability, and broad diet, has been increasingly recognized as a sentinel species for monitoring environmental pressures, including contaminants, emerging resistance, and zoonotic threats [23,32]. Our results demonstrate that red foxes can act as effective sentinels for antimicrobial resistance at the interface of wildlife, domestic animals, and humans. By reflecting resistance patterns associated with human-modified environments, foxes provide valuable insight into the occurrence of methicillin-resistant staphylococci and multidrug-resistant Gram-negative bacteria beyond that determined in clinical and agricultural settings. Incorporating this species into integrated surveillance frameworks could enhance early detection of emerging resistance patterns and inform strategies to protect public and animal health.

A major strength of this study lies in the combined use of two complementary phenotypic antimicrobial susceptibility testing methods, namely, disk diffusion (DD) and the Vitek 2 automated system. The high level of agreement observed between DD and Vitek 2 results for both Gram-negative and Gram-positive isolates supports the reliability of disk diffusion as a routine screening tool for antimicrobial resistance. This is particularly relevant in settings where access to automated systems such as Vitek 2 may be limited. Unlike automated platforms, disk diffusion allows greater flexibility in antibiotic selection and is not restricted to predefined testing panels, enabling the evaluation of a broader range of antimicrobials and enhancing the interpretative depth of resistance profiling. While Vitek 2 provides standardized minimum inhibitory concentration (MIC) values and remains a valuable method in clinical and research contexts, its narrower antibiotic coverage in this study (11 agents for Gram-negative and 14 for Gram-positive isolates) resulted in resistance data that was less comprehensive, as previously noted in other investigations [21,66]. Another important strength of this work is its contribution to the limited literature on antimicrobial resistance in Romanian wildlife, a field that remains underrepresented, particularly in Eastern Europe. By incorporating a comparative analysis between peri-urban and peri-rural environments, this study provides a nuanced dataset that enhances understanding of resistance patterns across differing ecological interfaces. Such data are essential for framing antimicrobial resistance within a One Health context, where interactions between wildlife, human activity, and environmental pressures play a critical role in resistance emergence and dissemination.

This study has several limitations that should be acknowledged. First, sampling was restricted to a single geographic area, encompassing three counties in western Romania, which may constrain the extrapolation of the findings to other regions of the country with different ecological or anthropogenic pressures. Second, although the study provided an in-depth characterization of phenotypic antimicrobial resistance patterns, molecular analyses of resistance determinants and clonal lineages were not performed. The inclusion of genotypic data would have enabled a more detailed understanding of the underlying resistance mechanisms and potential transmission pathways, particularly for clinically relevant determinants such as carbapenemase-encoding genes or methicillin resistance genes in *Staphylococcus aureus*. In this context, molecular confirmation could also have clarified the interpretation of isolates classified as susceptible with increased exposure by identifying cryptic resistance genes. Third, only a subset of isolates was examined using the Vitek 2 system due to practical constraints, which may introduce selection bias. Nevertheless, the sample size was sufficient for the statistical analyses performed and does not compromise the validity of the comparison of methods. This strategy was adopted due to practical and financial constraints and is now explicitly acknowledged as a limitation. Lastly, the absence of environmental sampling (e.g., soil and water) represents an additional limitation, as integrating such data would have provided a more comprehensive assessment of antimicrobial resistance across the animal–human–environment interface within a One Health framework.

These findings highlight the complexities of antimicrobial resistance in wildlife and point to key priorities for future research, including genetic analysis of resistance, tracing the sources of such resistance, and combining multiple testing methods to obtain a more complete understanding of resistance in the environment. Within this framework, the red fox appears as an important sentinel animal for monitoring antimicrobial-resistant bacteria in environments affected by anthropogenic activity. Integrated One Health initiatives informed by such surveillance may aid in the creation of evidence-based actions and preventative measures to reduce hazards to human, animal, and environmental health. Finally, continuous attention to animal sentinels may be critical for anticipating and resolving developing concerns at the human–environment interface.

## 4. Materials and Methods

### 4.1. Sample Source

**Figure 4:**
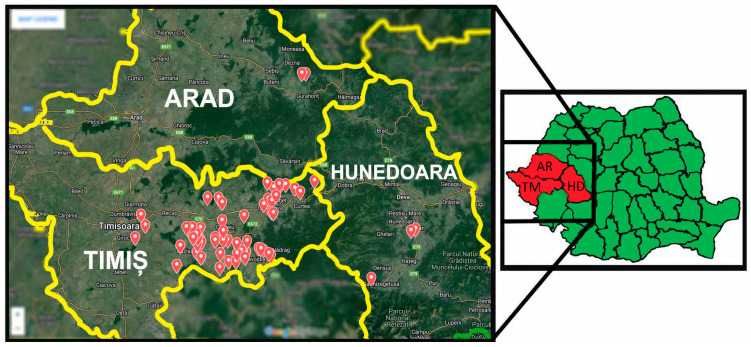
The GPS points where the fox carcasses were collected.

The research was conducted between 2022 and 2024, in western Romania, across the counties of Timiș (TM), Arad (AR) and Hunedoara (HD), during fox hunting periods established under Romania’s National Program for the Control, Surveillance, and Eradication of Rabies (Figure 4) [29,67]. The animals originated from multiple hunting grounds located in rural and semi-urban areas. These included Nădrag, Oloșag, Paniova, Făget, Traian Vuia, Sacoșul Mare, Boldur, Belinț-Chizătău, Buziaș, Moșnița, Visag, Valea Lungă, Margina, and Surduc. Additional samples were obtained from the Crocna-Zimbru hunting ground in Arad County, and from the Zeicani and Silvaș hunting grounds in Hunedoara County.

No ethical approval was required for this study, as all biological samples were obtained from red foxes legally hunted during officially established hunting seasons under the national program. Therefore, no animals were killed specifically for the purposes of this research [29,68].

Since Romania is not a rabies-free country, samples were collected, post mortem, from male and female foxes that were shot during hunting actions carried out under the national program for monitoring the effectiveness of anti-rabies vaccination [69]. Sampling was done at the Timiș County Veterinary and Food Safety Authority (D.S.V.S.A. Timiș) facility exclusively by qualified veterinary personnel, immediately after the carcasses had been received.

In total, rectal and oral swabs were collected from 108 red foxes (216 samples total) and processed using standard bacteriological methods. Swabs were aseptically inoculated into nutrient broth and incubated aerobically at 37 °C for 24 h for pre-enrichment. Cultures were then streaked onto selective and differential media: Eosin Methylene Blue (EMB) and Propylene glycol deoxycholate neutral red agar (Rambach) for Gram-negative bacteria, and Mannitol Salt Agar (MSA) and Baird-Parker agar for Gram-positive bacteria. Plates were incubated at 37 °C for 24–48 h [70,71].

Representative colonies were selected based on morphology, Gram-stained for preliminary classification, and transferred onto blood agar to obtain pure isolates. Final identification was performed using the Vitek^®^ 2 Compact system (bioMérieux, Marcy-l'Étoile, France), employing GN cards for Gram-negative bacteria and GP cards for Gram-positive bacteria, following the manufacturer’s instructions.

These samples were processed at the microbiology laboratory, where the behavior of the isolated pathogens against antimicrobial molecules was first tested using the Kirby–Bauer disk diffusion method, followed by the Vitek 2 Compact method.

### 4.2. Description of the Sampling Location

Firstly, depending on their human population density and proximity to urban or rural areas, the hunting grounds can be classified as peri-urban (high population density on the outskirts of cities, with high human activity), and peri-rural (lower population density, between rural villages, with agricultural or natural land use). In the first category, mention should be made of Buziaș, Moșnița, Făget, Traian Vuia and Margina, while the rest of the hunting grounds are associated with the latter category.

Secondly, depending on the presence or absence of water sources on their territory, the sampling locations can be classified as being without permanent water sources (dry terrain, forests), or with natural water sources (rivers, lakes). Therefore, in the first category are included the hunting grounds of Paniova, Silvaș and Sacoșul Mare, while the remaining ones are included in the second category.

### 4.3. The Antimicrobial Susceptibility of Bacterial Strains, Examined by Disk Diffusion Method

Antibiotic susceptibility testing was carried out using the Kirby–Bauer disk diffusion method using fresh cultures grown on Nutrient Agar for 18–24 h [13,72]. Inhibition zone diameters were interpreted in accordance with the clinical breakpoints and recommendations of the European Committee on Antimicrobial Susceptibility Testing (EUCAST) and Clinical and Laboratory Standards Institute (CLSI) [26,27] clinical breakpoints and recommendations. It should be mentioned that when EUCAST did not provide breakpoints for certain antimicrobials, the corresponding CLSI criteria were applied. Additional details on the methodology and reference values are provided in the Supplementary Material (Tables S1–S3).

To ensure accuracy, *Escherichia coli* ATCC 25922 and *Staphylococcus aureus* ATCC 25923 (Thermo Fisher Scientific, Lake Charles, LA, USA) were included as positive control strains, alongside a negative control.

Antibiotic susceptibility testing was performed on 137 bacterial strains, both Gram-negative and Gram-positive species. Among the Gram-negative bacteria, 73 strains were examined, including *Escherichia coli*, *Proteus mirabilis*, *Proteus vulgaris*, *Enterobacter* spp., *Salmonella enterica*, and *Shigella sonnei*. The Gram-positive group comprised 64 strains, represented by *Staphylococcus aureus*, *S. pseudintermedius*, *S. sciuri*, *S. vitulinus*, *S. xylosus*, *S. lentus*, *S. equorum*, *S. felis*, *S. cohnii* subsp. *cohnii*, *S. simulans*, *S. chromogenes*, and *S. warneri* (Table 15).

The susceptibility of Gram-negative strains was tested against 15 antibiotics belonging to the following antimicrobial groups: (I) Beta-lactams: Penicillins (Ampicillin, Amoxicillin), Cephalosporins (Ceftazidime, Cefoxitin, and Cephalexin), and Carbapenems (Imipenem); (II) Aminoglycosides: Gentamicin, Amikacin, and Tobramycin; (III) Tetracyclines: Tetracycline; (IV) Fluoroquinolones: Ciprofloxacin, Nalidixic acid; (V) Potentiated sulfonamides: Trimethoprim–Sulfamethoxazole; (VI) Nitrofurans: Nitrofurantoin; and (VII) Amphenicols: Chloramphenicol (Table 16 and Table S1).

Gram-positive strains were tested against 15 antibiotics grouped as follows: (I) Beta-lactams: Penicillin G, Oxacillin, Cefoxitin, Cephalexin, and Imipenem; (II) Aminoglycosides: Gentamicin, Amikacin, Tobramycin; (III) Tetracyclines: Tetracycline; (IV) Fluoroquinolones: Ciprofloxacin; (V) Potentiated sulfonamides: Sulfamethoxazole–Trimethoprim; (VI) Macrolides: Erythromycin; (VII) Lincosamides: Clindamycin; and (VIII) Amphenicols: Chloramphenicol (Table 16 and Table S1).

Bacterial strains were stored at minus 50 °C in brain–heart infusion broth (BHI) with glycerol. Prior to testing, subcultures were prepared on Müeller–Hinton Agar and incubated at 37 °C for 24 h. Suspensions were adjusted to a 0.5 McFarland standard and inoculated onto Müeller–Hinton agar plates. After maximum 15 min of calibration, the antimicrobial disks (Oxoid, Hampshire, UK) were applied onto the agar surface using a dispenser, followed by incubation at 37 °C for 24 h [13,72]. Inhibition zone diameters were measured, and isolates were classified as Susceptible (S), Resistant (R), or Susceptible at Increased Exposure (I), according to CLSI and EUCAST 2024 guidelines [26,27]. To ensure consistent interpretation of susceptibility data, a separate “IR” (intrinsic resistance) category was created for species with known intrinsic resistance and these were excluded from resistance-rate calculations, while isolates classified as “Susceptible, Increased Exposure” (I) were considered susceptible (S), in accordance with EUCAST (2024) and CLSI guidelines [26,27].

### 4.4. The Antimicrobial Susceptibility of Bacterial Strains, Examined by Vitek 2 Compact (BioMérieux, France)

Compared to the Kirby–Bauer, the Vitek 2 Compact system offers a more automated, quantitative and precise evaluation of antimicrobial resistance [21,66]. Out of the 137 strains, a subset of isolates initially tested by Kirby–Bauer was selected for Vitek 2 analysis. This subset included isolates initially classified as MDR, those resistant to at least two antibiotic classes, and isolates representing species for which limited data exist in the wildlife studies (e.g., *Proteus* spp., *Shigella sonnei*).

The subset included 41 Gram-negative strains, belonging to the species *E. coli* (n = 26), *P. mirabilis* (n = 10), *P. vulgaris* (n = 1), *S. sonnei* (n = 2) and *S. enterica* subsp. *enterica* (n = 2), along with 27 Gram-positive strains of the genus *Staphylococcus*, comprising the species *S. aureus* (n = 6), *S. pseudintermedius* (n = 6), *S. sciuri* (n = 6), *S. vitulinus* (n = 2), *S. xylosus* (n = 1), *S. lentus* (n = 3), *S. chromogenes* (n = 1) and *S. felis* (n = 2) (Table 17).

In order to determine the antimicrobial susceptibility of the Gram-negative species, the Vitek 2 AST-GN97 cards were used. These cards contain drugs which are used in both, veterinary and human medicine, such as (I) Beta-lactams: Penicillins (Ampicillin, Amoxicillin–Clavulanic acid), Cephalosporins (Cephalexin, Cefpodoxime), and Carbapenems (Imipenem); (II) Aminoglycosides: Amikacin, and Gentamicin; (III) Tetracyclines: Tetracycline, Doxycycline; (IV) Nitrofurans: Nitrofurantoin; (V) Amphenicols: Chloramphenicol; and (VI) Potentiated sulfonamides: Trimethoprim–Sulfamethoxazole. For doxycycline, the Advanced Expert System (AES) could not interpret the results, therefore, susceptibility was determined for only 11 antibiotics (Table S2) [21,66].

Regarding the Gram-positive isolates, the Vitek 2 AST GP80 cards were used, which include several antibiotics, out of which 14 reacted, as follows: (I) Beta-lactams: Oxacillin, Benzylpenicillin, Amoxicillin–Clavulanic acid, Cephalothin, and Cefoxitin; (II) Aminoglycosides: Gentamicin, Kanamycin; (III) Fluoroquinolones: Enrofloxacin, Marbofloxacin; (IV) Tetracyclines: Tetracycline; (V) Macrolides: Erythromycin; (VI) Lincosamides: Clindamycin; (VII) Potentiated sulfonamides: Trimethoprim–Sulfamethoxazole; and (VIII) Amphenicols: Chloramphenicol. In this case, cefoxitin is used for screening methicillin-resistant bacteria (Table S3) [66].

Following the manufacturer’s instructions, suspensions standardized to 0.5–0.63 McFarland turbidity scale were made using fresh bacterial cultures, and were loaded afterwards into the system tray. The Vitek 2 software automatically interprets the results in accordance with international standardized guidelines [66].

### 4.5. The Statistical Analysis

Equivalence between disk diffusion and Vitek 2 susceptibility testing was evaluated using McNemar’s test. Associations between isolate origin (peri-urban vs. peri-rural) and antimicrobial resistance profiles were assessed with the chi-square test, applying a significance threshold of *p* < 0.05. Fisher’s exact test was used when chi-square assumptions were not met.

## Figures and Tables

**Table 1:** Antimicrobial susceptibility of species isolated from the Buziaș, Moșnița, Făget, Traian Vuia and Margina hunting grounds.

Buziaș and Moșnița Hunting Grounds															
**Species**	Antimicrobial Substances														
	AMP	AML	CAZ	FOX	CL	IMI	CN	AK	TOB	TE	CIP	NA	SXT	F	C
*Escherichia coli*															
*Escherichia coli*															
*Escherichia coli*															
*Escherichia coli*															
*Escherichia coli*															
*Escherichia coli*															
*Escherichia coli*															
*Escherichia coli*															
*Escherichia coli*															
*Escherichia coli*															
*Escherichia coli*															
*Escherichia coli*															
*Escherichia coli*															
*Escherichia coli*															
*Escherichia coli*															
*Escherichia coli*															
*Enterobacter* spp.	IR	IR		IR	IR									IR	
*Proteus mirabilis*						IR				IR				IR	
*Proteus mirabilis*						IR				IR				IR	
*Proteus mirabilis*						IR				IR				IR	
*Salmonella enterica*														IR	
*Salmonella enterica*														IR	
Resistant%	38	38	50	29	14	37	41	45	36	16	36	9	9	0	14
Susceptible%	62	62	50	71	86	63	59	55	64	84	64	91	91	100	86
**Făget, Traian Vuia and Margina Hunting Grounds**															
Species	AMP	AML	CAZ	FOX	CL	IMI	CN	AK	TOB	TE	CIP	NA	SXT	F	C
*Escherichia coli*															
*Escherichia coli*															
*Escherichia coli*															
*Escherichia coli*															
*Escherichia coli*															
*Escherichia coli*															
*Escherichia coli*															
*Escherichia coli*															
Resistant%	25	25	12.5	50	12.5	12.5	37.5	50	50	25	37.5	12.5	0	12.5	0
Susceptible%	75	75	87.5	50	87.5	87.5	62.5	50	50	75	62.5	87.5	100	87.5	100

Legend: β-lactams: AMP (ampicillin), AML (amoxicillin), CAZ (ceftazidime), FOX (cefoxitin), CL (cephalexin), IMI (imipenem); aminoglycosides: CN (gentamicin), AK (amikacin), TOB (tobramycin); tetracyclines: TE (tetracycline); fluoroquinolones: CIP (ciprofloxacin), NA (nalidixic acid); potentiated sulfonamides: SXT (trimethoprim–sulfamethoxazole); nitrofurans: F (nitrofurantoin); amphenicols: C (chloramphenicol). Green = susceptibility to the tested antibiotic; Yellow = susceptibility at increased exposure (I); Red = resistance to the tested antibiotic; Gray (IR) = intrinsic resistance.

**Table 2:** Antimicrobial susceptibility of species isolated from the Oloșag, Boldur, Sacoșul Mare, Nădrag and Surduc hunting grounds.

Oloșag, Boldur and Sacoșul Mare Hunting Grounds															
Species	Antimicrobial Substances														
	AMP	AML	CAZ	FOX	CL	IMI	CN	AK	TOB	TE	CIP	NA	SXT	F	C
*Escherichia coli*															
*Escherichia coli*															
*Escherichia coli*															
*Escherichia coli*															
*Escherichia coli*															
*Escherichia coli*															
*Escherichia coli*															
*Escherichia coli*															
*Escherichia coli*															
*Escherichia coli*															
*Escherichia coli*															
*Escherichia coli*															
*Escherichia coli*															
*Escherichia coli*															
*Escherichia coli*															
*Proteus mirabilis*						IR				IR				IR	
*Proteus mirabilis*						IR				IR				IR	
*Proteus mirabilis*						IR				IR				IR	
*Proteus mirabilis*						IR				IR				IR	
*Proteus mirabilis*						IR				IR				IR	
*Proteus mirabilis*						IR				IR				IR	
*Shigella sonnei*														IR	
*Shigella sonnei*														IR	
Resistant%	26	26	30	17	13	35	13	22	13	29	17	9	9	7	9
Susceptible%	74	74	70	83	87	65	87	78	87	71	83	91	91	93	91
**Nădrag and Surduc Hunting Grounds**															
Species	AMP	AML	CAZ	FOX	CL	IMI	CN	AK	TOB	TE	CIP	NA	SXT	F	C
*Escherichia coli*															
*Escherichia coli*															
*Escherichia coli*															
*Escherichia coli*															
*Escherichia coli*															
*Escherichia coli*															
*Escherichia coli*															
*Escherichia coli*															
*Escherichia coli*															
*Proteus vulgaris*	IR	IR				IR				IR				IR	
Resistant%	56	67	70	30	50	44	40	50	40	33	50	30	10	11	30
Susceptible%	44	33	30	70	50	56	60	50	60	67	50	70	90	89	70

Legend: β-lactams: AMP (ampicillin), AML (amoxicillin), CAZ (ceftazidime), FOX (cefoxitin), CL (cephalexin), IMI (imipenem); aminoglycosides: CN (gentamicin), AK (amikacin), TOB (tobramycin); tetracyclines: TE (tetracycline); fluoroquinolones: CIP (ciprofloxacin), NA (nalidixic acid); potentiated sulfonamides: SXT (trimethoprim–sulfamethoxazole); nitrofurans: F (nitrofurantoin); amphenicols: C (chloramphenicol). Green = susceptibility to the tested antibiotic; Yellow = susceptibility at increased exposure (I); Red = resistance to the tested antibiotic; Gray (IR) = intrinsic resistance.

**Table 3:** Antimicrobial susceptibility of species isolated from the Belinț-Chizătău, Paniova, Valea Lungă, Silvaș and Zeicani hunting grounds.

Belinț-Chizătău, Paniova and Valea Lungă Hunting Grounds															
Species	Antimicrobial Substances														
	AMP	AML	CAZ	FOX	CL	IMI	CN	AK	TOB	TE	CIP	NA	SXT	F	C
*Escherichia coli*															
*Escherichia coli*															
*Escherichia coli*															
*Escherichia coli*															
*Proteus mirabilis*						IR				IR				IR	
Resistant%	20	20	60	20	40	40	40	40	40	60	60	20	20	20	40
Susceptible%	80	80	40	80	60	60	60	60	60	40	40	80	80	80	60
**Silvaș and Zeicani** **Hunting Grounds**															
Species	AMP	AML	CAZ	FOX	CL	IMI	CN	AK	TOB	TE	CIP	NA	SXT	F	C
*Escherichia coli*															
*Escherichia coli*															
*Escherichia coli*															
*Enterobacter* spp.	IR	IR		IR	IR									IR	
Resistant%	0	0	0	0	0	0	50	25	0	25	0	0	0	0	25
Susceptible%	100	100	100	100	100	100	50	75	100	75	100	100	100	100	75

Legend: β-lactams: AMP (ampicillin), AML (amoxicillin), CAZ (ceftazidime), FOX (cefoxitin), CL (cephalexin), IMI (imipenem); aminoglycosides: CN (gentamicin), AK (amikacin), TOB (tobramycin); tetracyclines: TE (tetracycline); fluoroquinolones: CIP (ciprofloxacin), NA (nalidixic acid); potentiated sulfonamides: SXT (trimethoprim–sulfamethoxazole); nitrofurans: F (nitrofurantoin); amphenicols: C (chloramphenicol). Green = susceptibility to the tested antibiotic; Yellow = susceptibility at increased exposure (I); Red = resistance to the tested antibiotic; Gray (IR) = intrinsic resistance.

**Table 4:** Antimicrobial susceptibility of species isolated from the Buziaș, Moșnița Făget, Traian Vuia and Margina hunting grounds.

Species	Buziaș and Moșnița Hunting Grounds													
	Antimicrobial Substances													
	OX	P	FOX	CL	IMI	CN	AK	TOB	TE	CIP	SXT	CD	E	C
*S. aureus*	N/A													
*S. aureus*	N/A													
*S. aureus*	N/A													
*S. aureus*	N/A													
*S. chromogenes*														
*S. cohnii cohnii*														
*S. cohnii cohnii*														
*S. lentus*														
*S. lentus*														
*S. pseudintermedius*														
*S. pseudintermedius*														
*S. sciuri*														
*S. sciuri*														
*S. sciuri*														
*S. sciuri*														
*S. vitulinus*														
*S. vitulinus*														
*S. xylosus*														
*S. xylosus*														
*S. xylosus*														
Resistant%	50	45	5	5	5	30	5	35	40	5	35	30	25	0
Susceptible%	50	55	95	95	95	70	95	65	60	95	65	70	75	100
**Făget, Traian Vuia and Margina Hunting Grounds**														
Species	OX	P	FOX	CL	IMI	CN	AK	TOB	TE	CIP	SXT	CD	E	C
*S. equorum*														
*S. sciuri*														
*S. sciuri*														
*S. sciuri*														
*S. sciuri*														
*S. vitulinus*														
*S. vitulinus*														
*S. vitulinus*														
Resistant%	50	50	0	0	0	12.5	12.5	12.5	37.5	12.5	25	37.5	0	0
Susceptible%	50	50	100	100	100	87.5	87.5	87.5	62.5	87.5	75	62.5	100	100

Legend: β-lactams: OX (oxacillin), P (penicillin G), FOX (cefoxitin), CL (cephalexin), IMI (imipenem); aminoglycosides: CN (gentamicin), AK (amikacin), TOB (tobramycin); tetracyclines: TE (tetracycline); fluoroquinolones: CIP (ciprofloxacin); sulfonamides: SXT (trimethoprim–sulfamethoxazole); lincosamides: CD (clindamycin); macrolides: E (erythromycin); amphenicols: C (chloramphenicol). Green = susceptibility to the tested antibiotic; Red = resistance to the tested antibiotic. Gray (N/A) = oxacillin susceptibility was not assessed for *S. aureus* by disk diffusion, in accordance with current guidelines.

**Table 5:** Antimicrobial susceptibility of species isolated from the Oloșag, Boldur, Sacoșul Mare, Nădrag and Surduc hunting grounds.

Species	Oloșag, Boldur, Sacoșul Mare Hunting Grounds													
	Antimicrobial Substances													
	OX	P	FOX	CL	IMI	CN	AK	TOB	TE	CIP	SXT	CD	E	C
*S. aureus*	N/A													
*S. aureus*	N/A													
*S. aureus*	N/A													
*S. aureus*	N/A													
*S. equorum*														
*S. equorum*														
*S. equorum*														
*S. felis*														
*S. lentus*														
*S. lentus*														
*S. lentus*														
*S. pseudintermedius*														
*S. pseudintermedius*														
*S. sciuri*														
*S. sciuri*														
*S. sciuri*														
*S. sciuri*														
*S. sciuri*														
*S. sciuri*														
*S. simulans*														
*S. vitulinus*														
*S. vitulinus*														
*S. warneri*														
*S. xylosus*														
Resistant%	40	45.8	8.3	8.3	12.5	25	4.2	29.2	29.2	8.3	12.5	33.3	4.2	4.2
Susceptible%	60	54.2	91.7	91.7	87.5	75	95.8	70.8	70.8	91.7	87.5	66.7	95.8	95.8
**Nădrag and Surduc Hunting Grounds**														
Species	Antimicrobial Substances													
	OX	P	FOX	CL	IMI	CN	AK	TOB	TE	CIP	SXT	CD	E	C
*S. felis*														
*S. felis*														
*S. lentus*														
*S. pseudintermedius*														
*S. sciuri*														
*S. vitulinus*														
Resistant%	33.3	33.3	16.7	16.7	16.7	50	0	50	66.7	66.7	50	83.3	16.7	0
Susceptible%	66.7	66.7	83.3	83.3	83.3	50	100	50	33.3	33.3	50	16.7	83.3	100

Legend: β-lactams: OX (oxacillin), P (penicillin G), FOX (cefoxitin), CL (cephalexin), IMI (imipenem); aminoglycosides: CN (gentamicin), AK (amikacin), TOB (tobramycin); tetracyclines: TE (tetracycline); fluoroquinolones: CIP (ciprofloxacin); sulfonamides: SXT (trimethoprim–sulfamethoxazole); lincosamides: CD (clindamycin); macrolides: E (erythromycin); amphenicols: C (chloramphenicol). Green = susceptibility to the tested antibiotic; Red = resistance to the tested antibiotic. Gray (N/A) = oxacillin susceptibility was not assessed for *S. aureus* by disk diffusion, in accordance with current guidelines.

**Table 6:** Antimicrobial susceptibility of species isolated from the Belinț-Chizătău, Silvaș, Zeicani and Crocna-Zimbru hunting grounds.

Species	Antimicrobial Substances													
	OX	P	FOX	CL	IMI	CN	AK	TOB	TE	CIP	SXT	CD	E	C
Belinț-Chizătău														
*S. vitulinus*														
Silvaș and Zeicani														
*S. sciuri*														
*S. vitulinus*														
*S. xylosus*														
Crocna-Zimbru														
*S. lentus*														
*S. xylosus*														

Legend: β-lactams: OX (oxacillin), P (penicillin G), FOX (cefoxitin), CL (cephalexin), IMI (imipenem); aminoglycosides: CN (gentamicin), AK (amikacin), TOB (tobramycin); tetracyclines: TE (tetracycline); fluoroquinolones: CIP (ciprofloxacin); sulfonamides: SXT (trimethoprim–sulfamethoxazole); lincosamides: CD (clindamycin); macrolides: E (erythromycin); amphenicols: C (chloramphenicol). Green = susceptibility to the tested antibiotic; Red = resistance to the tested antibiotic.

**Table 7:** Antimicrobial susceptibility of species isolated from the Buziaș, Moșnița, Făget, Traian Vuia and Margina hunting grounds.

Buziaș and Moșnița Hunting Grounds											
Species	Antimicrobial Substances										
	AMP	AMC	CL	CPD	IMI	AK	CN	TE	F	C	SXT
*Escherichia coli*											
*Escherichia coli*											
*Escherichia coli*											
*Escherichia coli*											
*Escherichia coli*											
*Escherichia coli*											
*Escherichia coli*											
*Escherichia coli*											
*Proteus mirabilis*					IR			IR	IR		
*Proteus mirabilis*					IR			IR	IR		
*Proteus mirabilis*					IR			IR	IR		
*Salmonella enterica*									IR		
*Salmonella enterica*									IR		
Resistant%	46	38	23	31	60	62	46	30	0	23	31
Susceptible%	54	62	77	69	40	38	54	70	100	77	69
**Făget, Traian Vuia and Margina Hunting Grounds**											
**Species**	AMP	AMC	CL	CPD	IMI	AK	CN	TE	F	C	SXT
*Escherichia coli*											
*Escherichia coli*											
*Escherichia coli*											
*Escherichia coli*											
*Escherichia coli*											
*Escherichia coli*											
*Escherichia coli*											
Resistant%	14	14	14	14	14	57	43	14	0	0	0
Susceptible%	86	86	86	86	86	43	57	86	100	100	100

Legend: β-lactams: AMP (ampicillin), AMC (amoxicillin–clavulanic acid), CL (cephalexin), CPD (cefpodoxime), IMI (imipenem); aminoglycosides: AK (amikacin), CN (gentamicin); tetracyclines: TE (tetracycline); nitrofurans: F (nitrofurantoin); amphenicols: C (chloramphenicol); potentiated sulfonamides: SXT (trimethoprim–sulfamethoxazole). Green = susceptibility to the tested antibiotic; Yellow = susceptibility at increased exposure (I); Red = resistance to the tested antibiotic; Gray (IR) = intrinsic resistance.

**Table 8:** Antimicrobial susceptibility of species isolated from the Oloșag, Boldur, Sacoșul Mare, Nădrag and Surduc hunting grounds.

Oloșag, Boldur and Sacoșul Mare Hunting Grounds											
Species	Antimicrobial Substances										
	AMP	AMC	CL	CPD	IMI	AK	CN	TE	F	C	SXT
*Escherichia coli*											
*Escherichia coli*											
*Escherichia coli*											
*Escherichia coli*											
*Escherichia coli*											
*Escherichia coli*											
*Proteus mirabilis*					IR			IR	IR		
*Proteus mirabilis*					IR			IR	IR		
*Proteus mirabilis*					IR			IR	IR		
*Proteus mirabilis*					IR			IR	IR		
*Proteus mirabilis*					IR			IR	IR		
*Proteus mirabilis*					IR			IR	IR		
*Shigella sonnei*									IR		
*Shigella sonnei*									IR		
Resistant%	36	21	21	7	63	21	14	25	17	7	14
Susceptible%	64	79	79	93	37	79	86	75	83	93	86
**Nădrag and Surduc Hunting Grounds**											
**Species**	**AMP**	**AMC**	**CL**	**CPD**	**IMI**	**AK**	**CN**	**TE**	**F**	**C**	**SXT**
*Escherichia coli*											
*Escherichia coli*											
*Escherichia coli*											
*Escherichia coli*											
*Proteus vulgaris*	IR	IR			IR			IR	IR		
Resistant%	75	75	80	40	75	80	80	75	0	40	20
Susceptible%	25	25	20	60	25	20	20	25	100	60	80

Legend: β-lactams: AMP (ampicillin), AMC (amoxicillin–clavulanic acid), CL (cephalexin), CPD (cefpodoxime), IMI (imipenem); aminoglycosides: AK (amikacin), CN (gentamicin); tetracyclines: TE (tetracycline); nitrofurans: F (nitrofurantoin); amphenicols: C (chloramphenicol); potentiated sulfonamides: SXT (trimethoprim–sulfamethoxazole). Green = susceptibility to the tested antibiotic; Yellow = susceptibility at increased exposure (I); Red = resistance to the tested antibiotic; Gray (IR) = intrinsic resistance.

**Table 9:** Antimicrobial susceptibility of species isolated from the Buziaș and Moșnița hunting grounds.

Buziaș and Moșnița Hunting Grounds														
Species	Antimicrobial Substances													
	FOX	OX	P	AMC	CEF	CN	K	ENR	MRB	TE	E	CD	SXT	C
*S. aureus*	NEG													
*S. aureus*	NEG													
*S. chromogenes*	NEG													
*S. lentus*	NEG													
*S. lentus*	NEG													
*S. pseudintermedius*	NEG													
*S. pseudintermedius*	POZ													
*S. pseudintermedius*	POZ													
*S. sciuri*	NEG													
*S. sciuri*	POZ													
Resistant%	30	30	70	50	30	30	10	10	10	40	30	50	70	0
Susceptible%	70	70	30	50	70	70	90	90	90	60	70	50	30	100

Legend: β-lactams: FOX (cefoxitin, used for screening methicillin-resistant bacteria; NEG = negative to screening/susceptible, POZ = positive/resistant), OX (oxacillin), P (benzylpenicillin), AMC (amoxicillin–clavulanic acid), CEF (cephalothin); aminoglycosides: CN (gentamicin), K (kanamycin); fluoroquinolones: ENR (enrofloxacin), MRB (marbofloxacin); tetracyclines: TE (tetracycline); macrolides: E (erythromycin); lincosamides: CD (clindamycin); potentiated sulfonamides: SXT (trimethoprim–sulfamethoxazole); amphenicols: C (chloramphenicol). Green = susceptibility to the tested antibiotic; Yellow = susceptibility at increased exposure (I); Red = resistance to the tested antibiotic.

**Table 10:** Antimicrobial susceptibility of species isolated from the Oloșag, Boldur, Sacoșul Mare, Nădrag and Surduc hunting grounds.

Oloșag, Boldur and Sacoșul Mare Hunting Grounds														
Species	FOX	OX	P	AMC	CEF	CN	K	ENR	MRB	TE	E	CD	SXT	C
*S. aureus*	POZ													
*S. aureus*	NEG													
*S. aureus*	POZ													
*S. aureus*	POZ													
*S. lentus*	NEG													
*S. pseudintermedius*	NEG													
*S. pseudintermedius*	POZ													
*S. sciuri*	POZ													
*S. sciuri*	POZ													
*S. xylosus*	NEG													
Resistant%	60	60	100	70	60	50	30	20	20	50	10	70	30	0
Susceptible%	40	40	0	30	40	50	70	80	80	50	90	30	70	100
**Nădrag and Surduc Hunting Grounds**														
**Species**	**FOX**	**OX**	**P**	**AMC**	**CEF**	**CN**	**K**	**ENR**	**MRB**	**TE**	**E**	**CD**	**SXT**	**C**
*S. felis*	NEG													
*S. felis*	NEG													
*S. pseudintermedius*	POZ													
*S. sciuri*	POZ													
*S. vitulinus*	NEG													
Resistant%	40	40	40	40	40	40	0	40	40	20	20	80	60	0
Susceptible%	60	60	60	60	60	60	100	60	60	80	80	20	40	100

Legend: β-lactams: FOX (cefoxitin, used for screening methicillin-resistant bacteria; NEG = negative to screening/susceptible, POZ = positive/resistant), OX (oxacillin), P (benzylpenicillin), AMC (amoxicillin–clavulanic acid), CEF (cephalothin); aminoglycosides: CN (gentamicin), K (kanamycin); fluoroquinolones: ENR (enrofloxacin), MRB (marbofloxacin); tetracyclines: TE (tetracycline); macrolides: E (erythromycin); lincosamides: CD (clindamycin); potentiated sulfonamides: SXT (trimethoprim–sulfamethoxazole); amphenicols: C (chloramphenicol). Green = susceptibility to the tested antibiotic; Yellow = susceptibility at increased exposure (I); Red = resistance to the tested antibiotic.

**Table 11:** Agreement between disk diffusion and Vitek 2 antimicrobial susceptibility results for Gram-negative strains.

Gram-Negative Strains Tested by Both Methods						c + d	*p*-Value (McNemar Test)
	Paired obs.	D_R_/V_R_ ^a^	D_S_/V_S_ ^b^	D_R_/V_S_ ^c^	D_S_/V_R_ ^d^		
Ampicillin AMP	40	16	23	1	0	1	1
Cephalexin CL	41	12	28	0	1	1	1
Ceftazidime CAZ/Cefpodoxime CPD	41	9	21	11	0	11	0.001
Imipenem IMI	30	15	12	3	0	3	0.25
Amikacin AK	41	21	18	2	0	2	0.5
Gentamicin CN	41	17	23	1	0	1	1
Tetracycline TE	30	7	20	3	0	3	0.25
Nitrofurantoin F	26	1	23	2	0	2	0.5
Chloramphenicol C	41	8	31	2	0	2	0.5
Trimethoprim-Sulfamethoxazole SXT	41	6	35	0	0	0	1

Legend: D_R_/V_R_ ^a^—no. of strains tested as resistant by both methods; D_S_/V_S_ ^b^—no. of strains tested as susceptible by both methods; D_R_/V_S_ ^c^—no. of strains tested as resistant by disk diffusion method and susceptible by Vitek 2; D_S_/V_R_ ^d^—no. of strains tested as susceptible by disk diffusion method and resistant by Vitek 2; c + d—sum of discordances.

**Table 12:** Agreement between disk diffusion and Vitek 2 antimicrobial susceptibility results for Gram-positive strains.

No. of Gram-Positive Strains Tested by Both Methods = 27					c + d	*p*-Value (McNemar Test)
	D_R_/V_R_ ^a^	D_S_/V_S_ ^b^	D_R_/V_S_ ^c^	D_S_/V_R_ ^d^		
Oxacillin OX *	7	4	8	2	10	0.11
Cefoxitin FOX	3	13	2	9	11	0.065
Gentamicin CN	11	12	2	2	4	1
Tetracycline TE	10	10	6	1	7	0.125
Trimethoprim-Sulfamethoxazole SXT	12	11	2	2	4	1
Clindamycin CD	15	4	5	3	8	0.727
Erythromycin E	5	20	1	1	2	1
Chloramphenicol C	0	27	0	0	0	N/A

Legend: *—21 strains tested for oxacillin susceptibility by both methods; D_R_/V_R_ ^a^—no. of strains tested as resistant by both methods; D_S_/V_S_ ^b^—no. of strains tested as susceptible by both methods; D_R_/V_S_ ^c^—no. of strains tested as resistant by disk diffusion method and susceptible by Vitek 2; D_S_/V_R_ ^d^—no. of strains tested as susceptible by disk diffusion method and resistant by Vitek 2; c + d—sum of discordances; N/A—statistical test not appliable because all the strains were susceptible by both methods.

**Table 13:** Comparison of antimicrobial susceptibility profiles of Gram-negative isolates from peri-urban and peri-rural areas, as determined using the disk diffusion method.

Gram-Negative Strains Tested by Disk-Diffusion Method (n = 73)						
Antimicrobials	Peri-Urban		Peri-Rural		chi-Value	*p*-Value
	R	S	R	S		
Ampicillin AMP ^a^	10	19	12	29	0.214	0.643
Amoxicillin AML ^a^	10	19	13	28	0.050	0.808
Ceftazidime CAZ	12	18	17	26	0.001	0.968
Cefoxitin FOX ^b^	10	19	8	34	2.159	0.142
Cephalexin CL ^b^	4	25	10	32	1.087	0.297
Imipenem IMI ^c^	8	19	11	24	0.023	0.879
Gentamicin CN	12	18	11	32	1.702	0.192
Amikacin AK	14	16	13	30	2.047	0.152
Tobramycin TOB	12	18	9	34	3.136	0.077
Tetracycline TE ^c^	5	22	11	24	1.326	0.249
Ciprofloxacin CIP	11	19	13	30	0.331	0.565
Nalidixic acid NA	3	27	7	36	0.589	0.443
Trimethoprim-Sulfamethoxazole SXT	2	28	4	39	0.162	0.687
Nitrofurantoin F ^d^	1	23	2	30	0.117	0.732
Chloramphenicol C	3	27	9	34	1.536	0.215

Legend: R = resistant; S = susceptible. Isolates exhibiting intrinsic resistance were excluded from the analysis as follows: ampicillin (^a^, n = 3), cefoxitin and cephalexin (^b^, n = 2), imipenem and tetracycline (^c^, n = 11), and nitrofurantoin (^d^, n = 17).

**Table 14:** Comparison of antimicrobial susceptibility profiles of Gram-negative isolates from peri-urban and peri-rural areas, as determined using the disk diffusion method.

Gram-Positive Strains Tested by Disk Diffusion Method (n = 64)						
Antimicrobials	Peri-Urban		Peri-Rural		chi-Value	*p*-Value
	R	S	R	S		
Penicillin G P	13	15	17	19	0.003	0.95
Oxacillin OX	12	12	14	18	0.215	0.643
Cefoxitin FOX	1	27	4	32	1.243	0.265
Cephalexin CL	1	27	4	32	1.243	0.265
Imipenem IMI	1	27	5	31	1.973	0.16
Gentamicin CN	7	21	10	26	0.062	0.803
Amikacin AK	2	26	2	34	0.067	0.795
Tobramycin TOB	8	20	12	24	0.166	0.683
Tetracycline TE	11	17	12	24	0.242	0.622
Ciprofloxacin CIP	2	26	7	29	1.972	0.16
Trimethoprim-Sulfamethoxazole SXT	9	19	7	29	1.354	0.244
Clindamycin CD	9	19	14	22	0.311	0.577
Erythromycin E	5	23	2	34	2.446	0.118
Chloramphenicol C	0	28	1	35	0.79	0.374

Legend: R = resistant; S = susceptible.

**Table 15:** Gram-negative and Gram-positive species tested for antimicrobial susceptibility with the disk diffusion method, and sampled on Müeller–Hinton Agar (Oxoid, Hampshire, UK).

Crt. No.	Bacterial Species	Strains No.	Sampling Place
Gram-negative			
1	*Escherichia coli*	56	rectal, oral
2	*Proteus mirabilis*	10	rectal
3	*Proteus vulgaris*	1	rectal
4	*Salmonella enterica*	2	rectal
5	*Shigella sonnei*	2	rectal
6	*Enterobacter* spp.	2	rectal
Total strains		73	
Gram-positive			
1	*Staphylococcus aureus*	8	rectal, oral
2	*Staphylococcus pseudintermedius*	7	rectal, oral
3	*Staphylococcus sciuri*	15	rectal, oral
4	*Staphylococcus vitulinus*	9	rectal, oral
5	*Staphylococcus lentus*	7	oral
6	*Staphylococcus xylosus*	6	rectal, oral
7	*Staphylococcus equorum*	4	rectal, oral
8	*Staphylococcus felis*	3	oral
9	*Staphylococcus cohnii cohnii*	2	rectal, oral
10	*Staphylococcus simulans*	1	rectal
11	*Staphylococcus chromogenes*	1	oral
12	*Staphylococcus warneri*	1	oral
Total strains		64	

**Table 16:** The antimicrobial substances tested on the Gram-negative and Gram-positive strains.

Antimicrobial Class	Antimicrobial Drug (µg)		Interpretation Reference
	Gram-Negative	Gram-Positive	
Beta-lactams	Ampicillin (10)	Penicillin G (10) Oxacillin (1) Cefoxitin (30) Cephalexin (30) Imipenem (10)	CLSI, 2024 EUCAST, 2024 [26,27]
	Amoxicillin (30)		
	Ceftazidime (10)		
	Cefoxitin (30)		
	Cephalexin (30)		
	Imipenem (10)		
Aminoglycosides	Gentamicin (10)	Gentamicin (10)	
	Amikacin (30)	Amikacin (30)	
	Tobramycin (10)	Tobramycin (10)	
Tetracycline	Tetracycline (30)	Tetracycline (30)	
Fluoroquinolones	Ciprofloxacin (5)	Ciprofloxacin (5)	
	Nalidixic acid (30)		
Potentiated sulfonamides	Trimethoprim– Sulfamethoxazole (25)	Trimethoprim– Sulfamethoxazole (25)	
Nitrofurans	Nitrofurantoin (100)	-	
Amphenicols	Chloramphenicol (30)	Chloramphenicol (30)	
Macrolides	-	Erythromycin (15)	
Lincosamides	-	Clindamycin (2)	

**Table 17:** Gram-negative and Gram-positive species tested for antimicrobial susceptibility with the Vitek 2 Compact System (BioMérieux, France).

Crt. No.	Bacterial Species	Strains No.	Sampling Place
Gram-negative			
1	*Escherichia coli*	26	rectal, oral
2	*Proteus mirabilis*	10	rectal
3	*Proteus vulgaris*	1	rectal
4	*Salmonella enterica*	2	rectal
5	*Shigella sonnei*	2	rectal
Total strains		41	
Gram-positive			
1	*Staphylococcus aureus*	6	rectal, oral
2	*Staphylococcus pseudintermedius*	6	rectal, oral
3	*Staphylococcus sciuri*	6	rectal, oral
4	*Staphylococcus vitulinus*	2	oral
5	*Staphylococcus lentus*	3	oral
6	*Staphylococcus xylosus*	1	rectal, oral
7	*Staphylococcus felis*	2	oral
8	*Staphylococcus chromogenes*	1	oral
Total strains		27	

## Data Availability

All data generated or analyzed during this study are included in the submitted version of the manuscript.

## Supplementary Materials

The following supporting information can be downloaded: https://www.mdpi.com/article/10.3390/antibiotics15020167/s1, Table S1:Antimicrobial substances used for Gram-negative and Gram-positive species with Disk Diffusimetry, Table S2: Antimicrobial substances used for Gram-negative species with Vitek-2 Compact system; Table S3: Antimicrobial substances used for Gram-positive species with Vitek-2 Compact system. References [26,27] are cited in the Supplementary Materials.

## Abbreviations

The following abbreviations are used in this manuscript:

MDR	Multidrug-resistant
IR	Intrinsic resistance
AMR	Antimicrobial resistance
EUCAST	European Committee on Antimicrobial Susceptibility Testing
ATCC	American Type Culture Collection
CLSI	Clinical and Laboratory Standards Institute
MRSA	Methicillin-resistant Staphylococcus aureus
MRS	Methicillin-resistant Staphylococcus
ESBL	Extended-spectrum β-lactamase
TM	Timiș county
HD	Hunedoara county
AR	Arad county
AMP	Ampicillin
AML	Amoxicillin
AMC	Amoxicillin–Clavulanic acid
P	Penicillin G
OX	Oxacillin
CAZ	Ceftazidime
CEF	Cephalothin
CL	Cephalexin
CPD	Cefpodoxime
FOX	Cefoxitin
IMI	Imipenem
CN	Gentamicin
AK	Amikacin
TOB	Tobramycin
TE	Tetracycline
DOX	Doxycycline
CIP	Ciprofloxacin
ENR	Enrofloxacin
MRB	Marbofloxacin
SXT	Trimethoprim–Sulfamethoxazole
F	Nitrofurantoin
C	Chloramphenicol
E	Erythromycin
CD	Clindamycin
K	Kanamycin
