# Supplementary material for: Phenotypic Antimicrobial Resistance of Some Bacterial Strains Isolated from Red Foxes (Vulpes vulpes) in Western Romania
Source: Antibiotics (Basel). 2026 Feb 4;15(2):167. doi: 10.3390/antibiotics15020167 (PMC12937231; doi:10.3390/antibiotics15020167)
Supplement: Supplementary file 1 [file antibiotics-15-00167-s001.zip › antibiotics-4095867-supplementary.pdf]

Supplementary Material

Table S1. Antimicrobial substances used for Gram-negative and Gram-positive species with Disk Diffusimetry

Antimicrobial substances	Code	Disk Content (μg)	Zone Diameter Breakpoint Limits (mm)		Interpretation Reference
Antimicrobial substances used for testing Gram-negative species					
			S ≥	R <	
Ampicillin	AMP	10	14	14	CLSI 2024 EUCAST 2024 [26, 27]
Amoxicillin	AML	30	14	14	
Ceftazidime	CAZ	10	22	19	
Cefoxitin	FOX	30	19	19	
Cephalexin	CL	30	14	14	
Imipenem	IMI	10	22	19	
Gentamicin	CN	10	17	17	
Amikacin	AK	30	18	18	
Tobramycin	TOB	10	16	16	
Tetracycline	TET	30	15	12	
Ciprofloxacin	CIP	5	25	22	
Nalidixic Acid	NA	30	19	14	
Sulfamethoxazole-Trimethoprim	SXT	25	14	11	
Nitrofurantoin	F	100	11	11	
Chloramphenicol	C	30	17	17	
**Antimicrobial substances used for testing Gram-positive species**					
Antimicrobial substances	Code	Disk Content (μg)	Zone Diameter Breakpoints Limits (mm)		Interpretation Reference
			S ≥	R <	
Penicillin G	P	10	29	29	CLSI 2024 EUCAST 2024 [26, 27]
Oxacillin	OX	1	20 (1[a])	20 (1[a])	
			18 (1[b])	18 (1[b])	
			25 (1[c])	25 (1[c])	
Cefoxitin	FOX	30	22 (2[a])	22 (2[a])	
			27 (2[b])	27 (2[b])	
			N/A (2[c])	N/A (2[c])	
Cephalexin	CL	30	N/A (3)	N/A (3)	
Imipenem	IMI	10	N/A (3)	N/A (3)	
Gentamicin	CN	10	18 (4[a])	18 (4[a])	
			22 (4[b])	22 (4[b])	
			15 (4[c])	13 (4[c])	
Amikacin	AK	30	15	15	
Tobramycin	TOB	10	18 (5[a])	18 (5[a])	
			20 (5[b])	20 (5[b])	
Tetracycline	TET	30	22	22	
Ciprofloxacin	CIP	5	21	16	
Sulfamethoxazole-Trimethoprim	SXT	25	17	14	
Chloramphenicol	C	30	18	13	
Erythromycin	E	15	21	21	
Clindamycin	CD	2	22	22	

Legend: (1[a], 1[b], 1[c]) = screening for methicillin resistance in *S. pseudintermedius*[1a], *S. intermedius*[1a], *S. schleiferi*[1a], *S. coagulans*[1a], *S. epidermidis*[1b], and other[1c] staphylococci except *S. aureus*; (2[a], 2[b], 2[c]) = susceptibility of cephalosporins is inferred from the cefoxitin susceptibility for *S. aureus*[2a], coagulase-negative staphylococci except *S. epidermidis*[2a], for *S. epidermidis*[2b], whereas for *S. pseudintermedius*[2c], *S. intermedius*[2c], *S. schleiferi*[2c], *S. coagulans*[2c] testing with oxacillin should be used instead; (3) = susceptibility is inferred from the cefoxitin susceptibility; (4[a], 4[b], 4[c]) = susceptibility to gentamicin for *S. aureus*[4a], coagulase-negative staphylococci[4b], and *S. pseudintermedius*[4c]; (5[a], 5[b]) = susceptibility to tobramycin for *S. aureus*[5a] and coagulase-negative staphylococci[5b].

Table S2. Antimicrobial substances used for Gram-negative species with Vitek-2 Compact system

Antimicrobial substances used for testing Gram-negative species (Vitek 2 AST-GN97 card)					
Antimicrobial substances	Code	Concentration (µg/ml)	Limits		FDA directions for use
			≤	≥	
Ampicillin	AM	4, 8, 32	2	32	CSAGNB**
Amoxicillin/ Clavulanic acid	AMC	4/2, 16/8, 32/16	2/1	32/16	CSAGNB**
Cephalexin	CN	8, 32, 64	4	64	N/A**
Cefpodoxime	CPD	0.5, 1, 4	0.25	8	CSAGNB**
Imipenem	IMI	1, 2, 6, 12	0.25	16	*Acinetobacter spp., Citrobacter spp., Enterobacter spp., E. coli, Klebsiella spp., M. morganii, P. vulgaris, Pv. rettgeri, P. aeruginosa, S. marcescens, Pv. stuartii*
Amikacin	AK	8, 16, 64	2	64	CSAGNB**
Gentamicin	CN	8, 32, 64	4	64	CSAGNB**
Tetracycline	TE	2, 4, 8	1	16	CSAGNB**
Doxycycline	DO	1, 4, 16	0.5	16	*Acinetobacter* spp., *E. aerogenes, E. coli, Klebsiella* spp., *Shigella* spp.
Nitrofurantoin	FT	16, 32, 64	16	512	CSAGNB**
Chloramphenicol	C	4, 16, 32	2	64	N/A**
Sulfamethoxazole–trimethoprim	SXT	1/19, 4/76, 16/304	20 (1/19)	320 (16/304)	*Klebsiella* spp., *Enterobacter* spp., *M. morganii, P. vulgaris, P. mirabilis, S. sonnei, S. flexneri*, Eco(+ETEC)**, *C. sakazakii*

Legend: FDA = Food and Drug Administration; **CSAGNB = clinical significance aerobic Gram-negative bacilli; **N/A = No specific FDA indications are recommended for use. **Eco (+ETEC) = *E. coli* (including sensitive enterotoxigenic strains involved in traveler's diarrhea).

Table S3. Antimicrobial substances used for Gram-positive species with Vitek-2 Compact system

Antimicrobial substances used for testing Gram-positive species (Vitek 2 AST-GP80 card)					
Antimicrobial substances	Code	Concentration (µg/ml)	Limits		FDA directions for use
			≤	≥	
Oxacillin	OX	0.5, 1, 2	0.25	4	*Staphylococcus* spp.
Benzylpenicillin (Penicillin G)	P	0.125, 0.25, 1, 2, 8, 64	0.12	64	*Enterococcus* spp.
			0.03	0.5	*Staphylococcus* spp.
			0.12	64	*S. agalactiae*
Amoxicillin/ Clavulanic acid	AMC	4/2, 8/4, 16/8	2 32	2 32	N/A**
Cefalothin	CF	4, 8, 32	2 32	2 32	N/A**
Cefoxitin (screening)	OXSF	6	NEG	POS	*Staphylococcus* spp.
Gentamicin	GM	8, 16, 64	0.5	16	*Staphylococcus* spp.

Kanamycin	K	32, 64, 128	4	64	N/A**
Enrofloxacin	ENR	1, 2	0.5	4	N/A**
Marbofloxacin	MRB	0.5, 2	0.5	4	N/A**
Tetracycline	TE	0.5, 1, 2	1	16	*Staphylococcus* spp., *Enterococcus* spp., *S. agalactiae*
Erythromycin	E	0.25, 0.5, 2	0.25	8	*Staphylococcus* spp., *Enterococcus* spp., *S. agalactiae*
Clindamycin	CM	0.06, 0.25, 1	0.125	4	MSSA**, MSSE**
Sulfamethoxazole–trimethoprim	SXT	8/152, 16/304, 32/608	10 (0.5/9.5)	320 (16/304)	N/A**
Chloramphenicol	C	2, 8, 16	4	64	*Staphylococcus* spp., *Enterococcus* spp., *S. agalactiae*

Legend: FDA = Food and Drug Administration; **N/A = No specific FDA indications are recommended for use; NEG = Negative; POS = Positive; **MSSA = Methicillin-susceptible *S. aureus*; **MSSE = Methicillin-susceptible *S. epidermidis*.
